# Conditional targeting of medium spiny neurons in the striatal matrix

**DOI:** 10.3389/fnbeh.2015.00071

**Published:** 2015-03-27

**Authors:** Björn Reinius, Martina Blunder, Frances M. Brett, Anders Eriksson, Kalicharan Patra, Jörgen Jonsson, Elena Jazin, Klas Kullander

**Affiliations:** ^1^Unit of Developmental Genetics, Department of Neuroscience, BMC, Uppsala UniversityUppsala, Sweden; ^2^Department of Organismal Biology, EBC, Uppsala UniversityUppsala, Sweden

**Keywords:** medium spiny neuron, matrix, striosome, patches, huntington's disease, behavior, SLC32A1, GABA

## Abstract

The striatum serves as the main input to the basal ganglia, and is key for the regulation of motor behaviors, compulsion, addiction, and various cognitive and emotional states. Its deterioration is associated with degenerative disorders such as Huntington's disease. Despite its apparent anatomical uniformity, it consists of intermingled cell populations, which have precluded straightforward anatomical sub-classifications adhering to functional dissections. Approximately 95% of the striatal neurons are inhibitory projection neurons termed medium spiny neurons (MSNs). They are commonly classified according to their expression of either dopamine receptor D1 or D2, which also determines their axonal projection patterns constituting the direct and indirect pathway in the basal ganglia. Immunohistochemical patterns have further indicated compartmentalization of the striatum to the striosomes and the surrounding matrix, which integrate MSNs of both the D1 and D2 type. Here, we present a transgenic mouse line, *Gpr101-Cre*, with Cre recombinase activity localized to matrix D1 and D2 MSNs. Using two *Gpr101-Cre* founder lines with different degrees of expression in the striatum, we conditionally deleted the vesicular inhibitory amino acid transporter (VIAAT), responsible for storage of GABA and glycine in synaptic vesicles. Partial ablation of VIAAT (in ~36% of MSNs) resulted in elevated locomotor activity compared to control mice, when provoked with the monoamine reuptake inhibitor cocaine. Near complete targeting of matrix MSNs led to profoundly changed motor behaviors, which increased in severity as the mice aged. Moreover, these mice had exaggerated muscle rigidity, retarded growth, increased rate of spontaneous deaths, and defective memory. Therefore, our data provide a link between dysfunctional GABA signaling of matrix MSNs to specific behavioral alterations, which are similar to the symptoms of Huntington's disease.

## Introduction

The striatum constitutes the largest component of the basal ganglia, integrating signals from the cerebral cortex, and is central for appropriate selection of behavioral action. Its dysfunction has been associated with classical motor disorders such as Parkinson's disease, Huntington's disease, and dystonia, but also with compulsivity, addiction, hyperactivity disorder, and depression (Hyman and Malenka, 2001; Yin and Knowlton, 2006; Crittenden and Graybiel, 2011; Shepherd, 2013). Yet, surprisingly little is known about its sub-compartmentalization and the behavioral correlates. The main barrier for such interrogation has been its apparent homogeneity, which obscures its inner compartments. The identification of two intermingled populations of MSNs, expressing either dopamine receptor D1 or D2 was an important step in our understanding of striatal subdivisions (Gerfen et al., 1990). The efferent projections of these molecularly defined populations constitute two separate pathways in the basal ganglia (Albin et al., 1989; Gerfen, 1992); a direct pathway (D1) projecting to the substantia nigra pars reticulata (SNr) and the internal globus pallidus (GPi), and an indirect pathway (D2) projecting to the external globus pallidus (GPe). Recently, the functional roles and precise distribution of these MSN sub-populations are beginning to become characterized in detail by use of the Cre/lox system (Bateup et al., 2010; Santini et al., 2012; Gangarossa et al., 2013; Rebholz et al., 2013; Arango-Lievano et al., 2014; Daigle et al., 2014).

A second level of sub-striatal segregation is that of the striatal patches and the surrounding matrix (Pert et al., 1976; Gerfen, 1984, 1992; Herkenham et al., 1984; Gerfen et al., 1985; Jimenez-Castellanos and Graybiel, 1989), also referred to as the striosome and the matrisome compartments (Crittenden and Graybiel, 2011). The first clue to this organization was the patchy enrichment of opiate receptors (Pert et al., 1976). Later, these zones were shown to be weak in acetylcholinesterase staining (Graybiel and Ragsdale, 1978; Herkenham and Pert, 1981). With the help of these and other molecular markers such as calbindin, that demarks the matrix (Gerfen et al., 1985), a coherent patch-matrix distribution in both the dorsal and the ventral striatum emerged (Gerfen, 1992).

The striatal patches receive most of their input from limbic regions, while the matrix receives input from the associative forebrain and sensorimotor cortex (Gerfen, 1984; Bolam et al., 1988; Ragsdale and Graybiel, 1988; McDonald, 1992; Flaherty and Graybiel, 1994). At the output side, both patch and matrix compartments participate in the direct and indirect pathways, but only patches project to the substantia nigra pars compacta (SNc) (Crittenden and Graybiel, 2011). These input and output divisions suggest that the activity of neurons located in patches or matrix promote different behaviors, but the lack of means to specifically target these compartments make their interrogation problematic, often relying on lesion studies. Transgenic mice are now emerging as useful tools to visualize these structures (Gerfen et al., 2013), including the Proenkephalin-eGFP mouse that preferentially label the matrix (Koshimizu et al., 2008), the Tyrosine Hydroxylase-eGFP (Miura et al., 2007) and Nr4a1-eGFP (Davis and Puhl, 2011) mice that mark the striosomes. Attempts to also investigate functions have used genetic manipulations, which have demonstrated that a selective change in the glutamate receptor-anchoring protein Homer in striosomes significantly affects motor performance (Tappe and Kuner, 2006).

Inhibitory neurotransmission in the adult nervous system is mainly mediated by release of γ-aminobutyric acid (GABA) and glycine from synaptic vesicles. The main vesicular transporter responsible for the filling of vesicles at GABAergic and glycinergic synapses is member 1 of the solute carrier family 32 (SLC32A1), also referred to as vesicular inhibitory amino acid transporter (VIAAT) or vesicular GABA transporter (VGAT). Whereas, GABAergic inhibition plays an essential role in the brain, both GABA and glycine act as the primary inhibitory neurotransmitters in the brainstem and spinal cord. Loss of GABA/glycine-mediated neurotransmission through deletion of VIAAT causes a drastic reduction of neurotransmitter release in both GABAergic and glycinergic neurons (Wojcik et al., 2006), which leads to embryonic lethality in null-mutants, presumably caused by omphalocele. Since constitutive *Viaat* knock-out (KO) mice die between E18.5 and birth, a conditional approach is required for analyzing the full spectrum of effects controlled by vesicular GABA and glycine release in the adult nervous system. Our group has previously addressed neuronal circuit functions in the mouse hippocampus using such an approach, where VIAAT mediated transmission was specifically removed from oriens lacunosum-moleculare cells through conditional deletion of VIAAT (Leao et al., 2012).

Here, we present the generation of a transgenic mouse line, *Gpr101-Cre*, which expresses Cre recombinase predominantly in matrix MSNs. Furthermore, by *Gpr101-Cre* mediated deletion of VIAAT we have identified behavioral changes that emerge from disruption of GABA signaling in neurons of the striatal matrix.

## Results

### Generation and characterization of Gpr101-Cre mice

**Figure 1:**
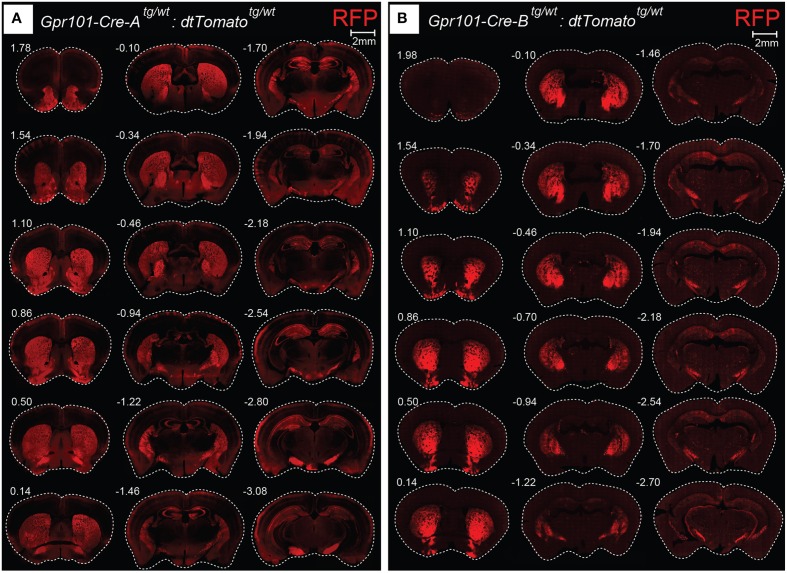
**Gpr101-Cre expression in the brain**. Fluorescence microscopy images showing coronal brain sections of **(A)** *Gpr101-Cre-A^tg/wt^:dtTomato^tg/wt^* and **(B)** *Gpr101-Cre-B^tg/wt^:dtTomato^tg/wt^* reporter mice. Numbers denote approximate bregma coordinates.

**Figure 2:**
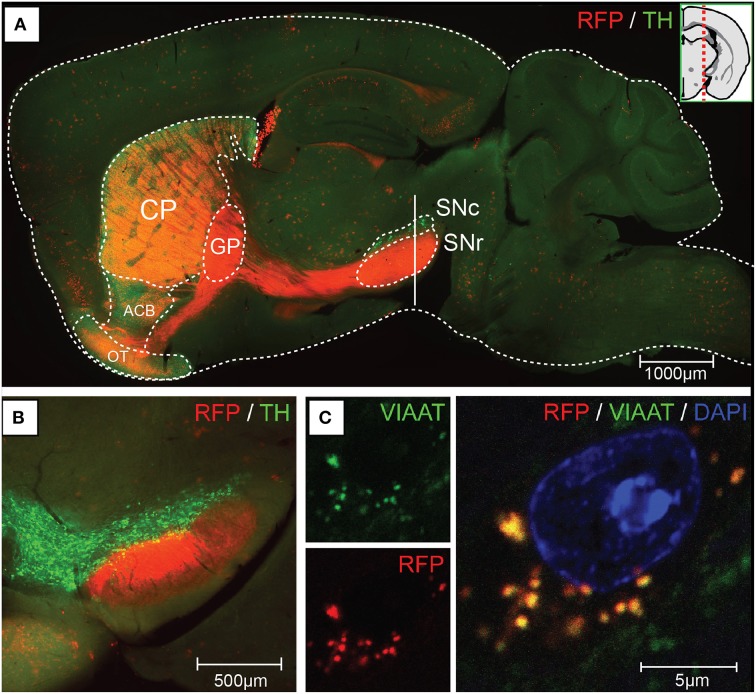
**Gpr101-Cre-B expression and projections**. Fluorescence microscopy images showing **(A)** a sagital brain section of *Gpr101-Cre-B^tg/wt^:dtTomato^tg/wt^* (RFP) combined with immunohistochemistry for Tyrosine hydroxylase (TH). **(B)** Coronal section at the vertical mark intersecting SN in **(A)** of *Gpr101-Cre-B^tg/wt^:dtTomato^tg/wt^* combined with immunohistochemistry for TH. **(C)** High resolution confocal microscopy image of *Gpr101-Cre-B^tg/wt^:dtTomato^tg/wt^* combined with immunohistochemistry for VIAAT, showing RFP/VIAAT positive terminals in the SNr.

We generated mice carrying Cre recombinase under the regulatory sequence of mouse *Gpr101*, an orphan receptor of the seven transmembrane domain type that is encoded in the X-chromosome, as described in detail in the Methods Section. In brief, we produced a linearized bacterial artificial chromosome (BAC) containing Cre in frame at the ATG site of *Gpr101*, flanked by 150 kbp of upstream and 30 kbp of downstream sequence (Figure S1). This BAC transgene was introduced into the genome of C57BL/6 by pronuclear injection, and resulted in the generation of two *Gpr101-Cre* positive founder individuals. We bred these founders on a C57BL/6 background, giving rise to two independently maintained *Grp101-Cre* lines, which we termed “*Grp101-Cre-A*” and “*Grp101-Cre-B*.” To investigate the activity of Cre in the mouse brain, we crossed the two *Gpr101-Cre* lines to the reporter *Gt(ROSA)26Sor*^*tm*14(*CAG*−*tdTomato*)*Hze*^ (*dtTomato*, Ai14) (Madisen et al., 2010). In the progeny of these mice, red fluorescent protein (RFP) is expressed in cells in which Cre has been active at any time during development or postnatal life. Most markedly, we observed RFP throughout the striatum of both *Gpr101-Cre* lines (Figure 1). Thus, an element within the *Gpr101-Cre* gene sequence drives striatal expression. *Gpr101-Cre-A* activated RFP was dense throughout the striatum, but also showed wider distribution of activity in other brain regions, such as the amygdala, hippocampus, hypothalamus, and in cortical layers (Figure 1A). While expression in the amygdala was found in a limited number of cells, the neuropil was giving a moderate red fluorescent signal. In the hippocampus, a fraction of pyramidal cells were labeled. *Gpr101-Cre-B*, on the other hand, showed fewer affected neurons within the striatum compared to *Gpr101-Cre-A*, but instead provided remarkable striatal specificity (Figure 1B). Intrigued by the restricted expression pattern, we characterized this founder line in more detail. The *Gpr101-Cre-B* activity was foremost concentrated to the caudate putamen (CP), nucleus accumbens (ACB), and olfactory tubercle (OT) (Figure 2A). We also observed limited expression in other brain regions including scattered hippocampal pyramidal neurons, neurons in the ventral retrosplenial area, scattered cortical neurons, and few cerebral neurons (Figure 2A, Figure S2). Additionally, we noticed red fluorescence in sporadic cells of arborescent appearance throughout the brain (Figure 2A, Figure S2). Some of these bush-like cells co-stained with antibodies for the glial fibrillary acidic protein (GFAP) whereas none co-stained with the neuronal marker NeuN (Figure S3), suggesting that these cells were astrocytes rather than neurons. In spinal cord sections, we found RFP labeling of sparse neurons in the dorsal horn and cells of astrocytic appearance throughout the gray commissure (Figure S4). Since the vast majority of all *Gpr101-Cre-B* labeled neurons were located in the striatum, we further investigated this population.

### Medium spiny neurons of both D1 and D2 type

**Figure 3:**
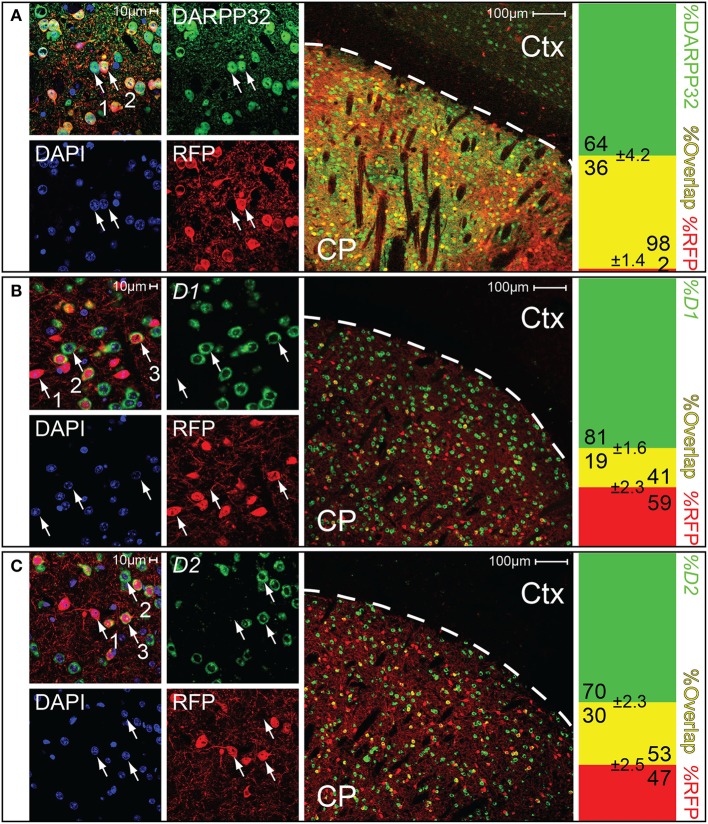
**Gpr101-Cre demarks medium spiny neurons of both D1 and D2 type**. Confocal microscopy images, showing brain sections of *Gpr101-Cre-B^tg/wt^:dtTomato^tg/wt^* combined with staining for either DARPP32 protein, *D1* mRNA, or *D2* mRNA. The leftmost panels show high resolution images in the striatum. The middle panels show wide-field images, CP: caudate putamen, Ctx: cortex. The rightmost panels show the percentage of cells with dual labeling (yellow) and single labeling (green or red) in cell counting experiments. #individuals = 2 and #sections = 10 per staining. **(A)** Immunohistchemical staining of the medium spiny neuron marker DARPP32 in *Gpr101-Cre-B^tg/wt^:dtTomato^tg/wt^*. (1) DARPP32^pos^:RFP^neg^ cell, (2) DARPP32^pos^:RFP^pos^ cell. **(B)** *In situ* hybridization of dopamine receptor 1 in *Gpr101-Cre-B^tg/wt^:dtTomato^tg/wt^*. (1) RFP^pos^:D1^neg^ cell, (2) RFP^neg^:D1^pos^ cell, (3) RFP^pos^:D1^pos^ cell. **(C)** *In situ* hybridization of dopamine receptor 2 in *Gpr101-Cre-B^tg/wt^:dtTomato^tg/wt^*. (1) RFP^pos^:D2^neg^ cell, (2) RFP^neg^:D2^pos^ cell, (3) RFP^pos^:D2^pos^ cell.

Medium spiny neurons (MSNs) are characterized by their expression of the dopamine- and cAMP-regulated phosphoprotein Mr~32,000 (DARPP32) (Ouimet and Greengard, 1990; Svenningsson et al., 2004; Matamales et al., 2009). Cell counting revealed that essentially all (~98%) of the *Gpr101-Cre-B* positive cells in the striatum co-stained with an antibody for DARPP32, and that around a third (~36%) of the DARPP32 stained cells were also *Gpr101-Cre-B* positive (Figure 3A). We found no *Gpr101-Cre-B* positive cell that co-labeled with choline acetyltransferase (ChAT). Moreover, *Gpr101-Cre-B* positive cells had dendrite formations and small soma diameters characteristic of MSNs (Figure S5) and distinct from striatal cholinergic interneurons (Figure S6). Therefore, we concluded that *Gpr101-Cre-B* delineates a population of MSNs in the striatum.

We next wanted to know whether *Gpr101-Cre-B* labeled MSNs of the direct pathway or the indirect pathway of the basal ganglia. Therefore, we performed staining experiments for dopamine D1 and D2 receptors, respectively. To label clearly distinguishable soma of dopamine receptor positive cells for counting, we performed fluorescent *in situ* hybridization of receptor *D1* or *D2* mRNA combined with immuno-labeling of *Gpr101-Cre-B* positive cells (we applied immuno-labeling of RFP, since the original reporter fluorescence was bleached by the *in situ* procedure). We found that ~41 and 53% of the *Gpr101-Cre-B* positive cells co-stained with *D1* and *D2* positive cells, respectively (Figures 3B,C). In further agreement with the conclusion that *Gpr101-Cre-B* marked MSNs of both the direct and the indirect pathway, we observed that *Gpr101-Cre-B* positive cells formed dense projections that terminated in both the GPi and GPe as well as in the SNr (Figure 2, Figure S2). GP and SNr were simultaneously deficient of RFP labeled soma (Figure S7), indicating that *Gpr101-Cre-B* cells project to, but not from, these nuclei. We therefore concluded that *Gpr101-Cre-B* marked a population of MSNs that include parts of both the direct and the indirect pathway of the basal ganglia.

### A marker for the striatal matrix

**Figure 4:**
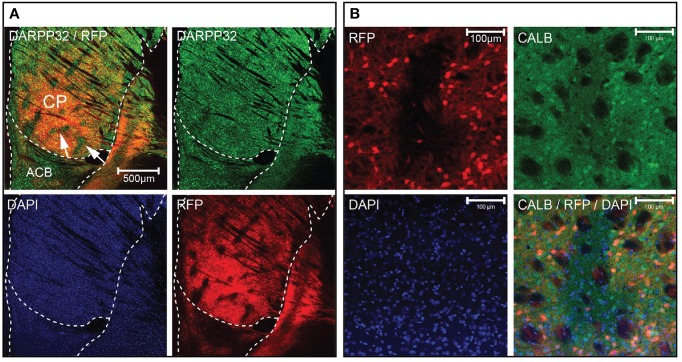
**Gpr101-Cre delineates the striatal matrix**. Confocal microscopy images showing **(A)** *Gpr101-Cre-B^tg/wt^:dtTomato^tg/wt^* combined with immunohistochemistry for DARPP32. The arrows point to patches in CP lacking RFP staining. Red: RFP, green: DARPP32, blue: DAPI. **(B)** *Gpr101-Cre-B^tg/wt^:dtTomato^tg/wt^* combined with immunohistochemistry for the matrix marker calbindin (CALB). Red: RFP, green: CALB, blue: DAPI.

We observed distinct unlabeled patches of tissue in the striatum of *Gpr101-Cre-B:dtTomato* mice (Figure 4A), reminiscent of the concept of a striatal mosaic (Gerfen, 1992). To investigate whether the lack of Cre activity in these regions correlated with the shape of striosomes, we performed calbindin immunohistochemistry. Calbindin 28K (CALB) is known to label the matrix, but show a diffuse decrease of labeling in striosomes (Gerfen et al., 1985; Difiglia et al., 1989; Jones, 1998; Matamales et al., 2009). Indeed, we found a decreased CALB immuno-stain in *Gpr101-Cre-B* deficient patches (Figure 4B). Previous studies have shown that matrix MSNs project to the SNr, whereas striosome MSNs project directly to the SNc (Gerfen, 1984; Fujiyama et al., 2011; Watabe-Uchida et al., 2012). In line with this, *Gpr101-Cre-B* terminals were enriched in the SNr but were not found in the SNc (Figures 2A,B, Figures S2, S7). Thus, the *Gpr101-Cre-B* mouse line provides a strategy to target and functionally interrogate MSNs of the striatal matrix, while avoiding striosomes.

### Phenotypic consequences upon deletion of VIAAT in the striatal matrix

**Figure 5:**
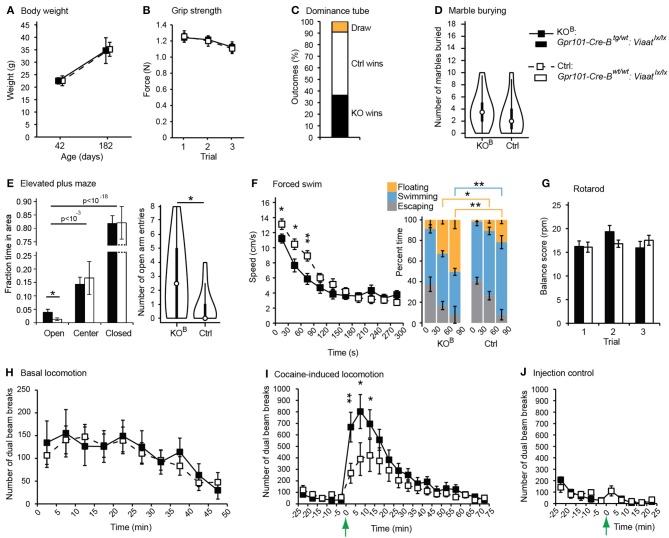
**Behavioral phenotypes upon partial matrix MSN VIAAT deletion**. Results from behavioral tests using *Gpr101-Cre-B^tg/wt^:Viaat^lx/lx^* (KO^B^, black) and *Gpr101-Cre-B^wt/wt^:Viaat^lx/lx^* (Ctrl, white). **(A)** Body weight at 6 and 26 wks. **(B)** Forelimb grip strength in three consecutive trials. **(C)** Dominance tube test. Proportion of wins. **(D)** Marble burying test. Number of marbles buried out of 18, shown as violin plots with the medians marked by circles. **(E)** Elevated plus maze. Proportion of time spent in areas during a 10 min trial, and number of open arm entries, shown as violin plots with the medians marked by circles. **(F)** Forced swim test. Average swim speed during 30 s segments of time over a 5 min trial. Barplots showing the percent time spent floating, swimming, and escaping (climbing against the cylinder wall), during the three first 30 s segments of time in the trial. **(G)** Rotarod test. Rotations per minute (rpm) at which the mice fell off the rotor as measured in three consecutive trials. **(H)** Basal locomotion. Number of dual beam breaks during 5 min segments of time over a 50 min trial. **(I)** Cocaine-induced locomotion. The basal locomotion was first monitored during 25 min, and at the time-point marked by the arrow 15 μg/g body weight of cocaine hydrochloride was injected. **(J)** Injection control. The mice were monitored during 25 min and injected with physiological saline solution at the time-point marked by the arrow (#KO^B^_inj−ctrl_ = 6, #Ctrl_inj−ctrl_ = 6). Bars and boxes show the group averages ±SEM. #KO^B^ = 11, #Ctrl = 11 if not stated otherwise. *P*-values: ^*^P < 0.05 and ^**^P < 0.01, according to two-sided *t*-tests [**A**, **B**, **E**(time), **F–J**]; two-sided binomial test (**C**); or two-sided Wilcoxon signed-rank test [**D**, **E**(entries)].

We wanted to identify behaviors that depend on correct signaling through the *Gpr101-Cre-B* positive population of MSNs. VIAAT, responsible for the loading of GABA and glycine into synaptic vesicles (McIntire et al., 1997; Sagne et al., 1997; Chaudhry et al., 1998), is the single member of its family and key for GABAergic transmission in the brain (Gasnier, 2004). As expected, *Gpr101-Cre-B* positive MSNs expressed *Viaat* mRNA (Figure S8), and VIAAT protein was found in *Gpr101-Cre-B* positive terminals (Figure 2C). We conditionally deleted VIAAT using our novel *Gpr101-Cre-B* Cre line and cross-bred these mice with mice carrying a conditional (floxed) *Viaat* allele (Tong et al., 2008). This produced *Gpr101-Cre-B^tg/wt^:Viaat^lx/lx^* knock-out (KO^B^) mice, in which *Viaat* was conditionally deleted (Figure S9), as well as *Gpr101-Cre-B^wt/wt^:Viaat^lx/lx^* littermate controls (Ctrls). We investigated these mice in a range of behavioral tests associated with diverse brain functions. Starting with the gross physiological parameters, KO^B^ mice did not differ from Ctrls in weight neither at the start of the trial (6 wks) nor at 26 wks (*P* > 0.05, *t*-test, Figure 5A). Also, they did not differ in their grip strength (*P* > 0.05, *t*-test, Figure 5B) or in the frequency of spontaneous deaths (zero spontaneous deaths at postnatal day 200 in both groups; *n* = 13 in each group). To test whether KO^B^ mice displayed changes that were reflected in their social status in the housing cages, we subjected weight-matched littermates to a dominance tube test. In this test, two mice were introduced in each end of a transparent tube with the approximate diameter of a mouse, so that when the mice met inside the tube one of them had to retreat by backing while the other one pushed forward (winner). Each KO^B^:Ctrl pair underwent four tube test trials, and mice with at least 3/4 wins were classified winners. There was no significant difference in the number of wins by KO^B^ and Ctrl (*P* > 0.05, binomial test, Figure 5C). We next applied the marble burying test to probe obsessive and repetitive perseverative behaviors. Each mouse was placed in a clean housing cage with 18 black glass marbles placed over a flat layer of sawdust bedding, and after 30 min in the arena the number of buried marbles was counted. This did not reveal any differences between the two groups (*P* > 0.05, Wilcoxon signed-rank test, Figure 5D). To test anxiety, fear, and exploratory behavior, we used an elevated plus maze. In both the KO^B^ and Ctrl group, we found that the mice avoided the open arm (*P* < 10^−3^ open *vs*. center, *P* < 10^−18^ open *vs*. closed, *t*-test), assuring that the open arm represented an adverse stimuli in our set-up. More interestingly, the KO^B^ mice spent more time in the open arm (*P* < 0.05, *t*-test), and re-entered the open arm more frequently (*P* < 0.05, Wilcoxon signed-rank test) than Ctrl mice (Figure 5E). To probe escape behavior and despair under an acutely stressful scenario, we performed a forced swim test. Each mouse was placed for 5 min in a transparent, water-filled, cylindrical container, from which they could not escape. The head of the mouse was tracked by computer software to follow their swim speed during the course of the experiment. This showed that KO^B^ mice produced significantly less movement than the Ctrls during the initial 90 seconds into the trial (*P* < 0.01, *t*-test, Figure 5F), while after around 3 min, both groups converged at the same constant speed. We measured the time that the mice spent swimming, floating, or escaping/climbing against the cylinder wall, during the initial 90 s of the forced swim test and found that KO^B^ and Ctrl spent similar amount of time trying to escape, but KO^B^ mice spent significantly less time swimming and more time floating (*P* < 0.01, *t*-test, Figure 5F). To test whether KO^B^ mice had altered sensorimotor coordination or balance ability, we subjected each mouse to three consecutive trials in the rotarod test, but found no difference in any of the three trials (*P* > 0.05, *t*-test, Figure 5G). The striatum is crucial for the regulating voluntary movement, and ablation of the central signaling protein DARPP32 in D1 and D2 MSNs have been demonstrated to have opposite effects on locomotor behavior (Bateup et al., 2010), decreasing or increasing basal locomotion, respectively. Hence, we tracked spontaneous movement in an open field chamber. In this test, KO^B^ and Ctrl mice presented equal basal locomotor activity (*P* > 0.05, *t*-test, Figure 5H). However, when the mice were provoked with the monoamine reuptake inhibitor cocaine, which induces locomotion, we found a larger cocaine-induced locomotion increase in KO^B^ mice compared to Ctrls (*P* < 0.01, *t*-test, Figures 5I,J). This showed that the consecutive loss of VIAAT in the *Gpr101-Cre-B* activated cells has consequences for signaling and functions associated with the dopamine system.

**Figure 6:**
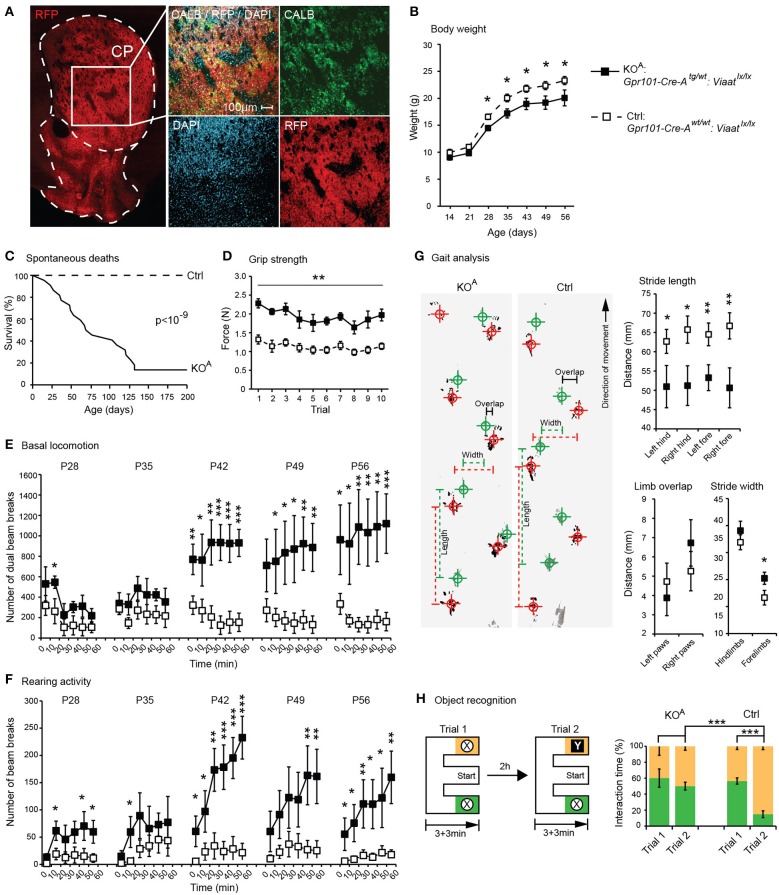
**Behavioral phenotypes upon broad matrix MSN VIAAT deletion**. Results from CALB staining and behavioral tests using *Gpr101-Cre-A^tg/wt^:Viaat^lx/lx^* (KO^A^, black) and *Gpr101-Cre-A^wt/wt^:Viaat^lx/lx^* (Ctrl, white). **(A)** Confocal microscopy images showing *Gpr101-Cre-A^tg/wt^:dtTomato^tg/wt^* combined with immunohistochemistry for CALB. **(B)** Body weights up to adult age. **(C)** Survivability during 200 days (#KO^A^ = 22, #Ctrl = 24), *P*-value according to Fischer's exact test at day 200. **(D)** Forelimb grip strength in 10 consecutive trials. **(E)** Basal locomotion. Number of dual beam breaks during 10 min segments of time over a 60 min trial. **(F)** Rearing activity. Number of beam breaks during 10 min segments of time over a 60 min trial. **(G)** Gait analysis. Footprints recorded from KO^A^ and Ctrl mice during locomotion (hind paws: green, fore paws: red) and summary statistics. **(H)** Object recognition test. A schematic illustration of the paradigm, and the proportion of time spent interacting with objects. Bars and boxes show the group averages ±SEM. #KO^A^ = 5, #Ctrl = 6. *P*-values: ^*^P < 0.05, ^**^P < 0.01, ^***^P < 0.001, according to two-sided *t*-tests if not stated otherwise.

Consequently, we concluded that GABA signaling of matrix MSNs has a role in the regulation of locomotion. We reasoned that since *Gpr101-Cre-B* did not target all matrix MSNs, but only ~36% (Figure 3A), adaptive compensation of the remaining untargeted MSNs might have prevented the manifestation of more severe locomotion phenotypes. To evaluate this possibility, we turned to the *Gpr101-Cre-A* line with its broader expression. CALB staining on *Gpr101-Cre-A:dtTomato* mice showed that also this line expressed Cre in the striatal matrix (Figure 6A), but incorporated virtually all matrix MSNs in contrast to the more limited targeting in *Gpr101-Cre-B*. Occasionally, some few cells were found in the border zone between the matrix and striosome in the *Gpr101-Cre-A*, something we did not observe in the *Gpr101-Cre-B*. When handling *Gpr101-Cre-A^tg/wt^:Viaat^lx/lx^* (KO^A^) mice, we soon noticed that these mice were slightly smaller compared to their littermate controls, and we therefore monitored their weights through postnatal development. This showed that KO^A^ and controls gained weight at a similar rate during the two first weeks of life, but after this point KO^A^ mice showed arrested growth that persisted into adulthood (*P* < 0.05, *t*-test, Figure 6B). Moreover, KO^A^ mice had severely decreased survivability (*P* < 10^−9^, Fischer's exact test, Figure 6C).

We also noticed that KO^A^ mice walked with jerky or somewhat stiff limb movements. Grip strength tests indicated that KO^A^ mice had exaggerated muscle rigidity (*P* < 0.01, *t*-test, Figure 6D). We monitored basal locomotion in KO^A^ and Ctrls from age P28 and 5 weeks onwards, and found that as the KO^A^ mice aged they developed increasingly exaggerated locomotor behaviors (*P* < 0.001, *t*-test, Figure 6E), and presented rearing behaviors not seen in control mice (*P* < 0.001, *t*-test, Figure 6F). To further investigate the KO^A^ locomotor phenotype, we analyzed their gait letting the mice traverse a 1 m × 50 mm track and recording their footfall patterns on absorbent paper (Figure 6G). This test demonstrated that KO^A^ mice walked with significantly shorter strides (*P* < 0.01, *t*-test) and had slightly wider spacing between the forelimbs (*P* < 0.05, *t*-test).

In addition to its role in regulating locomotor functions, the striatum is known to participate in a variety of cognitive functions, including working memory (Lovinger, 2010; Palmiter, 2011). Notably, we also found some *Gpr101-Cre-A* activity in the hippocampus, which is well-known to control memory function. However, this Cre expression was limited to excitatory pyramidal neurons that are unlikely to be affected by the deletion of VIAAT. We tested memory in KO^A^ mice in a novel object recognition paradigm using an E-maze setup (Figure 6H). Whereas control mice showed strong preference for interacting with novel objects, KO^A^ mice did not discriminate between the familiar and the novel object (*P* < 0.001, *t*-test, on the difference in preference).

## Discussion

Here we present the effects of conditionally deleting the GABA presynaptic vesicular transporter VIAAT in MSNs of the striatal matrix. We characterized the novel Cre line *Gpr101-Cre-B* and found that it predominantly expressed active Cre protein in MSNs of both the D1 and D2 type in the striatal matrix, and had projections to the GPe, GPi, and SNr. Deletion of VIAAT in limited parts of the matrix (~36% of MSNs), driven by the *Gpr101-Cre-B* mouse line, had mild effects with no major changes in weight, motor abilities, spontaneous locomotion, defensive or social behaviors. In contrast, we observed a reduced level of anxiety, as measured by the increased number of both entries and time spent in the open arm of the elevated plus maze. Moreover, and perhaps most striking, *Gpr101-Cre-B^tg/wt^:Viaat^lx/lx^* mice were hyper-responsive to cocaine-induced locomotion (Figure 5I). The VIAAT lox allele has been successfully used in several previous studies (e.g., Leao et al., 2012; Rahman et al., 2014), hence, phenotypic effects in *Gpr101-Cre-B^tg/wt^:Viaat^lx/lx^* mice were expected. Consequently, we found that loss of VIAAT in the *Gpr101-Cre-A* line, with its broader expression incorporating virtually all matrix MSNs, resulted in more severe phenotypes, including shorter life span, locomotor, and cognitive defects (Figure 6). Clearly, the more limited deletion was not sufficient to reveal these stronger defects and could be compensated for, likely by the remaining MSNs in the *Gpr101-Cre-B* mouse line.

However, we also find it plausible that other factors may have influenced the results, such as other neurotransmitters compensating for the loss of VIAAT or rearrangement of alternative neuronal circuits regulating the observed behaviors in the *Gpr101-Cre-A-* as well as the *B*-line. Currently, we have not quantified the reduction of GABA release; such analyses will aid in the interpretation of the consequences of VIAAT deletion. Circuit rearrangements are certainly possible in light of data suggesting the presence of subspecific afferent and efferent connections to the matrix and the striosomes. MSNs in the matrix, but not in the striosomes, receive input from ontogenetically younger cortical layers, such as the upper layer V in the somatosensory cortex (Wilson, 1987; Gerfen, 1989; Saka and Graybiel, 2003; Crittenden and Graybiel, 2011). Moreover, there is also evidence of a striosome specific pathway to the substantia nigra pars compacta (Gerfen, 1984; Jimenez-Castellanos and Graybiel, 1989; Tokuno et al., 2002; Fujiyama et al., 2011), suggesting that striosomal MSNs are in a position to control the flow of dopamine to the striatum coming from neurons of the SNc (Crittenden and Graybiel, 2011). In support of this, we found that the projections from *Gpr101-Cre-B* labeled MSNs were absent in the substantia nigra pars compacta (Figure 2, Figure S2). Thus, loss of a major transmitter in the matrix neurons would not affect the direct feedback of dopamine from the pars compacta. More likely, this would lead to consequences associated with matrix function, influenced by afferent projections from the somatosensory, motor, and association cortices and less influence of the limbic system influenced by afferents innervating striosomes from the orbitofrontal, anterior cingulate, and insular cortices (Ragsdale and Graybiel, 1990; Flaherty and Graybiel, 1994; Eblen and Graybiel, 1995; Kincaid and Wilson, 1996; Levesque and Parent, 1998). Hence, to more fully understand the participation of the MSNs in striatal related functions, the *Gpr101-Cre-A-* and *B*-line should be used in temporally controlled experiments i.e., using a conditionally activated diphtheria toxin receptor.

Despite possible compensatory mechanisms, the severe phenotypes in the *Gpr101-Cre-A* line makes it worthwhile to compare the observed symptoms with those found in basal ganglia disorders such as Huntington's disease. Typically, such patients loose their ability to control their movements and display jerky, random, and uncontrollable movements called chorea (Walker, 2007). Patients have a shorter life expectancy at least in part resulting from poor muscle coordination, difficulty clearing the lungs and an increased risk of aspirating food or drink resulting in eating difficulties and malnutrition (Aziz et al., 2008). Moreover, cognitive disabilities are common and as the disease progresses, deficits in short- and long-term memory appear (Montoya et al., 2006). Interestingly, the phenotypes of the *Gpr101-Cre-A:Viaat* KO mice recapitulate some of these symptoms, having a shorter life span, locomotor deficits and impaired memory function (Figure 6). We speculate that the disease progression in humans and the compensatory mechanisms upholding normal function to a certain extent in the mouse model, tentatively explains the similar phenotypes. Although the exact molecular mechanism has to be worked out, we find the apparent similarities as a reasonable starting point for the further investigation of matrix MSNs.

The deletion of VIAAT does not include all matrix MSNs in the *Gpr101-Cre-B* mice, about 36% of them are affected as estimated by recombination of the *tdTomato* allele (Figure 3A). Nevertheless, we could observe alterations on locomotor response induced by cocaine, which would be in line with the expected role of matrix MSNs based on the inputs from the motor cortex to the matrix. However, we also observed small but significant effects on behaviors associated to the limbic system, since the elevated plus maze and the porsolt forced swim test are primarily designed to measure anxiety and depression-like states. This questions whether there exists a functional selectivity for patch and matrix compartments for regulation of particular behaviors. A caveat to this reasoning is the inherent problem with analysis of anxiety-like behavior based on observation of motor activity. Thus, the observed differences in behaviors in the elevated plus maze or the porsolt swim test might also be due to alterations in motor circuits. Moreover, it should be noted that differences in the novel object recognition test using the *Gpr101-Cre-A* line might have been influenced by possible deletion of VIAAT in a limited number of cells in the amygdala, or in the border zone between the striosome and matrix compartments (Figure 1A).

Although no previous studies have investigated a matrix-specific loss of neurotransmitter signaling, comparisons can be made with mice that have lost MSN functionality. DARPP32, a crucial component of protein kinase and phosphatase signaling in MSNs, is essential for biochemical and behavioral responses in the striatum (Svenningsson et al., 2004). Thus, deletion of DARPP32 will hamper the functionality of the targeted pathway, and since DARPP32 is expressed in virtually all MSNs, a dysfunctional protein will affect both the striatonigral and striatopallidal projection pathways. Mice that lack DARPP32 show an attenuated locomotor responsiveness to a single dose of injected cocaine (Fienberg et al., 1998), suggesting that the MSN population as a whole become less sensitive to dopamine, and as a result the mice become hypo-locomotive. In contrast, the increased locomotor response to cocaine in *Gpr101-Cre-B^tg/wt^:Viaat^lx/lx^* mice suggests that the targeted MSNs are distorting the balance of inhibitory actions between MSN subpopulations. We thus speculate that under normal conditions, matrix MSNs, or a subpopulation thereof, are involved in reducing locomotor activity. It has previously been suggested that increased striosome activity promotes stereotypies whereas matrix activity permits flexibility in motor behaviors (Crittenden and Graybiel, 2011). Our findings here are just beginning to shed some light on matrix MSN functionalities, and clearly, more detailed experiments are required to corroborate their functional role, for example by investigating the responses of *Gpr101-Cre-B^tg/wt^:Viaat^lx/lx^* mice to other psychostimulant drugs.

Nevertheless, this is the first example of a study that specifically targets the signaling of matrix MSNs. As such, it represents a significant advance in the understanding of how the neurocircuitry of the basal ganglia is functionally organized. Further studies of matrix neurons, for example through optogenetic approaches, are now feasible and will be important for a detailed understanding of the functional division of the striatal patches and matrix.

## Methods

### Experimental animals

The studies were approved by the regional ethical committee on animal experiments (Swedish Board of Agriculture, Jordbruksverket, permit: C79/9). The mouse lines used were: *Gt(ROSA)26Sor^tm14(CAG−tdTomato)Hze^* (*tdTomato*, Ai14) (Madisen et al., 2010), *Viaat-lx-lx* (exon 2 of the *Slc32a1* gene flanked by loxP sites) (Tong et al., 2008), and *Gpr101-Cre-A/B* (herein described). Mice were bred on C57BL/6 background and housed at the animal facility of Uppsala Biomedical Center (Uppsala University).

### Generation of *Gpr101-Cre* mice

The *Gpr101-Cre* construct was generated by recombineering (Lee et al., 2001), introducing the Cre gene at the ATG site in the first coding exon (exon 2) of the *Gpr101* gene in a BAC. The BAC covered approximately 150 kbp of genomic sequence upstream of the *Gpr101* start site, and 30 kbp of sequence downstream of the of the *Gpr101* gene. An overview of the recombineering procedure is shown in Figure S1. We first generated a linear DNA containing *nls*Cre-SV40polyA-FRT-Kan/Neo-FRT, flanked by two 50 bp homologous to sequences upstream or downstream of *Gpr101* exon 2 (Details available upon request). The BAC (RP23-203E19, Chloramphenicol^R^, ChrX: 54711000-54916304) was electroporated into EL250 cells, and seeded on an LB agarose plate (chloramphenicol 12.5 μg/ml). Successful introduction of RP23-203E19 was confirmed by colony PCR using the primers 5′-GAAGATAGCCACCCTGACCTT and 5′-TGCTCTTGTGTTCTGTTTTTGTG. Localizations of all primers used for the colony PCRs are shown in Figure S1B. Next, the *nls*Cre-SV40polyA-FRT-Kan/Neo-FRT construct was inserted into exon 2 of *Gpr101* in the BAC by homologous recombination, mediated by EL250 endogenous recombination proteins (Lee et al., 2001). Successful introduction of the Cre construct into the BAC was confirmed by colony PCR using the primers 5′-GCACAGGGATCTGAGAAAGG and 5′-AGGCAAATTTTGGTGTACGG (detecting Cre insertion), and the primers 5′-TCGCCTTCTTGACGAGTTCT and 5′-TGCTCTTGTGTTCTGTTTTTGTG (detecting kanamycin insertion) on chloramphenicol/kanamycin double resistant clones (LB agar plates, chloramphenicol 12.5 μg/ml; kanamycin 25 μg/ml). The kanamycin selection marker was subsequently deleted by activation of EL250 indigenous Flp recombinases. This was confirmed using the primers 5′-TGCTGGAAGATGGCGATTAG and 5′-CCCTTTGCCTGAAATGGTAA. Sequencing of the BAC construct was performed using the primers 5′-TCCTCTGCAAGGCACTAACC, 5′-CTGATTTCGACCAGGTTCGT, 5′-ATACCGGAGATCATGCAAGC, 5′-GACCGGCAAACGGACAGAAG, 5′-CTGACCAGAGTCATCCTTAGC, and 5′-TTATTTATCTGCCGTGTGGTGG. The modified BAC was linearized by cleavage with NotI, and the backbone of the BAC (containing a chloramphenicol resistance gene) was removed by size-dependent fractionation through a custom-made sepharose column. Fractions were inspected using Pulse Field Gel Electrophoresis (CHEF mapper, Bio-Rad, CA) (Figure S1C). The *Grp101-Cre* DNA construct was introduced randomly into the mouse genome by pronuclear injection (Uppsala University Transgenic Facility, Department of Medical Biochemistry and Microbiology, BMC). The progeny was genotyped by PCR on tail biopsies, using the primers: 5′-GCACAGGGATCTGAGAAAGG and 5′-AGGCAAATTTTGGTGTACGG, and resulted in the identification of 2 founder individuals (Figure S1D).

### Genotyping

Mice were genotyped by PCR using tail biopsies, with the following primers. *Gpr101-Cre:* 5′-GCACAGGGATCTGAGAAAGG and 5′-AGGCAAATTTTGGTGTACGG. *Viaat-lx-lx:* 5′-TCCTTTGTGGCTTCCTTCCG (common forward primer), 5′-GGATAGAAGAAGTGTGGACC (gives differently sized bands for wild-type *Viaat* and intact *Viaat-lx-lx* alleles), and 5′-GCAGTGGACCTTGGATGTCTATC (specific to excised *Viaat*^lx*/lx*^ alleles). *tdTomato:* 5′-CTGTTCCTGTACGGCATGG, 5′-GGCATTAAAGCAGCGTATCC, 5′-AAGGGAGCTGCAGTGGAGTA, and 5′-CCGAAAATCTGTGGGAAGTC.

### Tissue preparation

Mice used for *in situ* hybridization and immunohistochemistry were anesthetized by intraperitoneal (IP) injection of 1:1 Domitor (70 μg/g bodyweight, Orion) and Ketalar (7 μg/g bodyweight, Pfitzer) and transcardially perfused with PBS (0.1 M Na_2_PO_4_, pH 7.4) followed by 4% formaldehyde. Whole dissected brains were further fixed at 4°C overnight (spinal cords for ~4 h) in 4% formaldehyde. The brains were sectioned to 60 μm using a vibratome (Leica, Germany).

### Immunohistochemistry

The sections were rinsed with PBS and blocked in 0.3% Trition X-100 in PBS with either 3% goat serum or a commercial blocking reagent (Roche Diagnostics), for 60-90 min. Primary antibodies were diluted in blocking buffer and incubated over night at 4°C. List of antibodies, their suppliers and concentrations: NeuN (Chemicon, Cat. MAB377) 1:400, ChAT (Chemicon, Cat. AB143) 1:100, DARPP32 (Novus Biologicals, Cat. NB100-79931) 1:800, GFAP (Sigma-Aldrich, Cat. G3893) 1:1000, VIAAT (Synaptic Systems, Cat. 131-011) 1:400, TH (Chemicon, Cat. AB152) 1:400, CALB (Swant, Cat. CB38) 1:10000. The sections were washed in PBS and incubated with the appropriate secondary antibodies (conjugated to Alexa488 or Alexa568 (Invitrogen)) and DAPI (Sigma-Aldrich) 1:1000 in PBS or PBT for 2–4 h at room temperature. Immunolabeled sections were washed in PBT (0.1% Tween 20 in PBS) and 3-4 rounds of PBS, and mounted on glass slides using Mowiol (Sigma-Aldrich). Minor modifications to the incubations and washes were implemented to optimize the conditions for each individual antibody. Immuno- and reporter RFP signals were detected using a fluorescence microscope (BX61WI, Olympus) or a confocal microscope (LSM 510 META, Zeiss). Images were stacked using ImageJ (v1.46, NIH). Brightness and contrast were uniformly adjusted using Photoshop CS3 (Adobe), and pictures consisting of several overlapping image frames were merged using the function Photomerge (Photoshop CS3). Animals used for immunohistochemistry were naive to behavioral tests and VIAAT KO.

### Combined *in situ* hybridization and immunohistochemistry

Antisense and sense *in situ* hybridization probes were generated by *in vitro* transcription of a linearized plasmid containing cDNA sequence (*D1, D2*, or *Viaat* obtained from Invitrogen) using T7, SP6 or T3 polymerase and labeled with digoxygenin (Roche Applied Science) according to the manufacturer's instructions. Sections for *in situ* hybridization were bleached in 6% hydrogen peroxide for 15 min at room temperature, followed by washes in PBT, and then 0.5% Triton X-100 in PBS. After cleansing in PBS, sections were digested with 20 μg/ml Proteinase K (Invitrogen), and postfixed in 4% formaldehyde before preincubation in hybridization buffer (50% formamide, 5 × SSC, pH 4.5, 1% sodium dodecyl sulfate, 50 μg/ml tRNA, 50 μg/ml heparin in PBT). Hybridization was carried out over night at 58°C. To remove unbound probes, the sections were sequentially incubated in a wash buffer (50% formamide, 5 × SSC, pH 4.5, and 1% SDS in PBT), followed by incubation in a second wash buffer (50% formamide, 2 × SSC, pH 4.5, in PBT) at 58°C. The sections were cleansed in PBS, and treated with a commercial blocking buffer (Roche Diagnostics). Sections were incubated overnight at 4°C together with anti-digoxigenin alkaline phosphatase-conjugated antibody diluted 1:5000 in blocking buffer (Roche Diagnostics). Signal augmentation was achieved by the use of Fast Red tablets (Roche Diagnostics). After development, the tissues were washed in PBT supplemented with 0.1%, MP (Biomedicals). To visualize bleached RFP (since the *in situ* procedure incapacitates the original RFP fluorescence) from the tomato positive cells the sections were stained with a rabbit antibody to RFP (Abcam, 1:200). Sections were incubated with a secondary antibody [Alexa488-conjugated donkey antibody to rabbit (Invitrogen) 1:400]. Sections were washed in PBS to remove excessive secondary antibodies and mounted on glass slides using Mowiol (Sigma-Aldrich). The slides were scanned using a Zeiss LSM 510 META confocal microscope and stacked using ImageJ (v1.46, NIH). Brightness and contrast were uniformly adjusted using Photoshop CS3 (Adobe). Animals used for *in situ* hybridization were naive to behavioral tests and VIAAT KO.

### Cell counting and size measurements

Cell counting for the overlap between *Gpr101-Cre-B^tg/wt^:dtTomato^tg/wt^* and DARPP32, *D1* and *D2* mRNA was performed in ImageJ (v1.46, NIH) by manual counting on confocal microscopy images (acquired as described in a previous section), using five sections from two different individuals for each staining (#_sections total_ = 10 per staining). Measurements of soma and nuclei diameters were also performed on confocal images using ImageJ (v1.46, NIH).

### Behavioral tests

The *Grip strength* was measured by putting the mouse forepaws on a wire mesh (BIO-GS3, Bioseb), and slowly pulling the mouse back from the device by a tail grip until the animal released the wire mesh. The *Marble burying test* (Deacon, 2006) was performed by placing 18 black glass marbles in a grid pattern on top of a 5 cm layer of sawdust bedding in a clean housing cage, and counting the number of completely buried marbles after a mouse had spent 30 min in such an arena. The *Elevated plus maze* (Walf and Frye, 2007) was situated 52 cm above the floor, and consisted of four arms of which two had walls and two were open. The mouse was placed in the center of the maze facing toward one of the closed arms, and its activity during 10 min in the maze was video recorded. The illumination at the maze was maintained at 100 lux. AniTracker (rsutils) was used for the analysis of the recorded data. *Forced swim tests* (Porsolt et al., 1977; Petit-Demouliere et al., 2005) were executed in a Plexiglas cylinder (20 cm in diameter), filled up to 25 cm depth with water (25°C). The trial lasted for 5 min and was video recorded from above, and the movement of the head during swimming was tracked using custom software. The fraction time spent swimming, escaping, or floating was manually scored during the first 90 s of video recordings using custom software that recorded the time that keyboard buttons (representing the three behaviors) were pressed. In the *Rotarod test* (Dunham and Miya, 1957) the speed of the rotor (IITC Life Science) was linearly increased from 0 to 45 rpm over 60 s, and the rpm at which the mouse fell from the rotating cylinder was recorded. Spontaneous and cocaine-induced *Locomotion* were tracked using an open field chamber that was gridded by infrared beams and detectors, which automatically counted the number of dual beam breaks per time unit. KO and Ctrl mice of matched body weight were tested simultaneously in identical open field chambers. The dose of cocaine was 15 μg/g body weight, and IP injected. *Rearing activity* was measured using the same device as for locomotion, but with a beam grid located further up from the floor of the arena. For the *Gait analysis*, the mice were trained to walk along a 1 m x 50 mm track with 15 cm high walls, 1 day prior to testing. A bright light source was placed at the start site and an escape box was located at the end of the track to encourage locomotion. Initially, the mice were held in supine restraint and their paws were mock-painted using a paintbrush. They were then placed at the start of the track and had to traverse to the end without stopping at least 3 times. During actual testing, the procedure was repeated, but the hind paws were painted blue and the forepaws red (Sense finger paints, Försäljning AB, Sweden). Footprints were recorded on chromatography paper with 3–4 runs per mouse. Gait patterns were scanned as an image, crosshairs were placed at each paw mark, and stride length, limb overlap, and stride width were analyzed. The E-maze used for the *Object recognition test* was constructed from black plastic walls and a white inset floor (Overall size: 400 × 250 × 210 mm). The familiar objects were metal cones and the novel object was a clean glass salt and pepper shaker with a metal lid. The objects were approximately 100 mm high × 50 mm in diameter. Exploration and interaction with the objects were recorded by a ceiling-mounted video camera and manually scored using AniTracker (rsutils). Mice were placed in the start arm (center) and habituated to the empty arena for 3 min. They were then removed from the maze and placed into a holding cage while two familiar objects were placed in the outer arms. The mice were then returned to the start arm (center) and allowed to explore for 3 min. At trial completion, the mice were returned to the home cage for 2 h before the test was repeated; 3 min of habituation, followed by 3 min of exploration of a familiar and novel object. The time spent interacting with each object (being within 2 cm distance) was recorded. Animals were used in multiple behavioral tests, performed in the following order for the KO^B^/KO^B^-Ctrl cohort (≥P56, and at least 3 days between each test): grip strength, dominance tube, marble burying, elevated plus maze, rotarod, forced swim, basal locomotion, cocaine-induced locomotion, and injection control (#KO^B^ = 11, #Ctrl = 11 for all test except the injection control in which #KO^B^ = 6, #Ctrl = 6). Behavioral tests for the KO^A^/KO^A^-Ctrl cohort were performed at the following postnatal days: basal locomotion and rearing activity (P28, P35, P42, P49, P56), gait analysis (P60-61), grip strength (P64), object recognition (P68) (#KO^A^ = 5, #Ctrl = 6). Arenas and equipment that were not completely replaced between trials were washed with mild detergent and 70% ethanol to minimize interference between consecutive behavioral tests. Any behavioral tests that required manual scoring were performed blind to the mouse genotype.

### Statistical analyses

Statistical significance of phenotypic differences between KO and Ctrl mice were assessed in R (R Core Team, 2014) using the following tests: Student's two-sided *t*-test [Body weight, grip strength, elevated plus maze (proportion of time spent in areas)], forced swim test, rotarod test, locomotion (basal, cocaine-induced, and injection control), rearing activity, gait analysis, and object recognition test; two-sided Wilcoxon signed-rank test [Elevated plus maze (number of entries) and marble burying test]; two-sided binominal test (Dominance tube test); Fisher's exact test (Survivability).

## Author contributions

Generated the Cre lines: BR. Characterized Cre expression and performed immunohistochemical experiments: BR. Participated in the *in situ* hybridization experiments: BR and AE. Performed the behavioral experiments on *Gpr101-Cre-B*: BR with assistance of KP. Performed the behavioral experiments on *Gpr101-Cre-A*: FB with assistance of MB. Wrote the paper: BR and KK, with contributions from JJ, FB, and MB. Conceived the study: KK, EJ, and BR. Planned the experiments: KK, EJ, FB, MB, and BR. Contributed material and reagents: KK and EJ.

### Conflict of interest statement

The authors declare that the research was conducted in the absence of any commercial or financial relationships that could be construed as a potential conflict of interest.
