# Supplementary material for: Conditional targeting of medium spiny neurons in the striatal matrix
Source: Front Behav Neurosci. 2015 Mar 27;9:71. doi: 10.3389/fnbeh.2015.00071 (PMC4375991; doi:10.3389/fnbeh.2015.00071)

Supplementary Figures for

Conditional targeting of medium spiny neurons in the striatal matrix

Björn Reinius[1,2], Martina Blunder[1], Frances M. Brett[1], Anders Eriksson[1],

Kalicharan Patra[1], Jörgen Jonsson[1], Elena Jazin[2], and Klas Kullander[1,*]

[1]Department of Neuroscience, BMC, Uppsala University, Sweden

[2]Department of Organismal Biology, EBC, Uppsala University, Sweden

[*]Corresponding author: klas.kullander@neuro.uu.se

1. Supplementary Figure Legends

Figure S1. Generation of Gpr101-Cre mice

Schematic illustration of the recombination technique. **(A)** A linear dsDNA fragment, encoding *nls*Cre-SV40polyA-FRT-Kan/Neo-FRT, flanked on each side by 50 bp sequences homologous to the 50 bp sequences upstream and downstream of exon 2 in the *Gpr101*, was introduced into EL250 cells. The *Gpr101*-containing BAC (RP23-203E19, Chloroamphenicol^{R}, Chr.X: 54711000- 54916304) was electroporated into these cells, in which recombination took place. The kanamycin selection marker was deleted by activation of EL250 indigenous Flp recombinases. **(B)** The success of each step during construction was controlled using the primers represented by arrows in the figure (described in detail in the Methods section). **(C)** The modified BAC was linearized by cleavage with NotI, and the backbone of the BAC was removed by size-dependent fractionation on a sepharose column. These fractions were inspected by pulse field gel electrophoresis, as shown in the figure. The green arrow shows the position of the backbone fragment and the red arrow the position of the insert. The asterisk on top of fraction 10 and 11 indicates that these fractions were used for pronuclear injection. **(D)** The progeny were analyzed for the presence of the *Gpr101-Cre* transgene by PCR on tail biopsies. The figure shows the bands for two founders, which gave rise to the *Gpr101-Cre-A* and *Gpr101-Cre-B* lines.

Figure S2. Characterization of Gpr101-Cre-B expression in the brain

Fluorescence microscopy images, showing sagital brain sections of *Gpr101-Cre-B*$^{tg/wt}$*:dtTomato*$^{tg/wt}$ combined with immunohistochemistry for either **(A-I)** TH or **(J-O)** DARPP32. Red: RFP, green: TH or DARPP32.

Figure S3. Arborescent cells

Confocal microscopy images of *Gpr101-Cre-B*$^{tg/wt}$*:dtTomato*$^{tg/wt}$ showing cells of arborescent appearance in the hippocampus, combined with immunohistochemistry labeling of either **(A)** the astrocytic marker GFAP or **(B)** the neuron marker NeuN. Red: RFP, green: GFAP or NeuN, gray: DAPI.

Figure S4. Characterization of Gpr101-Cre-B in the spinal cord

Fluorescence microscopy images, showing spinal cord sections of *Gpr101-Cre-B*$^{tg/wt}$*:dtTomato*$^{tg/wt}$.

Figure S5. Size distribution of matrix MSNs

Histograms showing the size distribution of measured soma and nucleus diameter of RFP and DARPP32 positive cells in the striatum ($\#_{cells}$=120/staining; *Gpr101-Cre-B*$^{tg/wt}$*:dtTomato*$^{tg/wt}$). The mean and standard deviation (sd) is given above each histogram.

Figure S6. ChAT immunohistochemistry

Confocal microscopy images from the striatum of *Gpr101-Cre-B*$^{tg/wt}$*:dtTomato*$^{tg/wt}$ combined with immunohistochemistry for ChAT. Red: RFP, green: ChAT, gray: DAPI.

Figure S7. Efferent projections to GP and SNr

Confocal microscopy images from the caudate putamen (CP), external / internal globus pallidus (GPe/GPi), and substantia nigra reticulata (SNr); showing RFP positive projections, but the lack of RFP positive soma in GP and SNr.

Figure S8. Viaat mRNA expression in Cre positive soma

Confocal microscopy images from the CP of *Gpr101-Cre-B*$^{tg/wt}$*:dtTomato*$^{tg/wt}$. tdTomato (RFP) immunohistochemistry combined with *in situ* hybridization for *Viaat* mRNA. Red: RFP, green: *Viaat* mRNA, blue: DAPI.

Figure S9. Gpr101-Cre mediates flox-out of the conditional Viaat allele

Gel electrophoresis of PCR products amplified from striatal gDNA. A combination of three PCR primers, giving products of different sizes for the wildtype allele (green), the intact allele *Viaat-lx-lx* (blue), and the excised *Viaat-lx-lx* allele (red), were used to test whether the *Gpr101-Cre* mediated flox-out of the *Viaat*$^{lx/lx}$ allele occured. *Gpr101-Cre*$^{tg/wt}$*:Viaat*$^{lx/lx}$ mice (the first five lanes to the left) presented both excised and intact *Viaat-lx-lx* alleles, as expected from conditional knock-out.

Figure S1

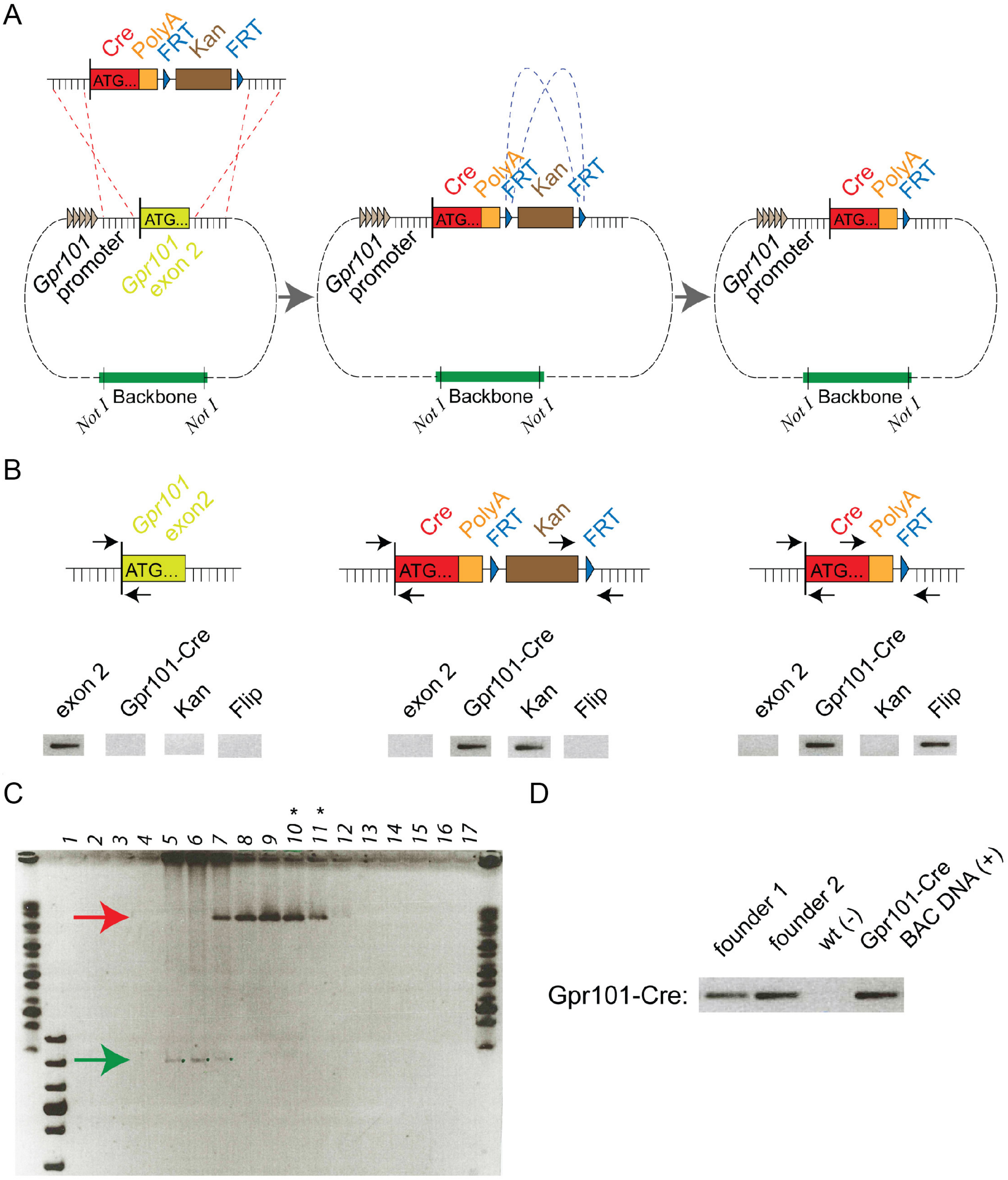

A
Cre
PolyA
FRT
Kan
FRT
ATG...
Gpr101 promoter
Gpr101 exon 2
Backbone
Not I
Not I
B
Gpr101 exon2
exon 2
Gpr101-Cre
Kan
Flip
C
1 2 3 4 5 6 7 8 9 10 * 11 * 12 13 14 15 16 17
D
founder 1
founder 2
wt (-)
Gpr101-Cre BAC DNA (+)
Gpr101-Cre:

Figure S2A

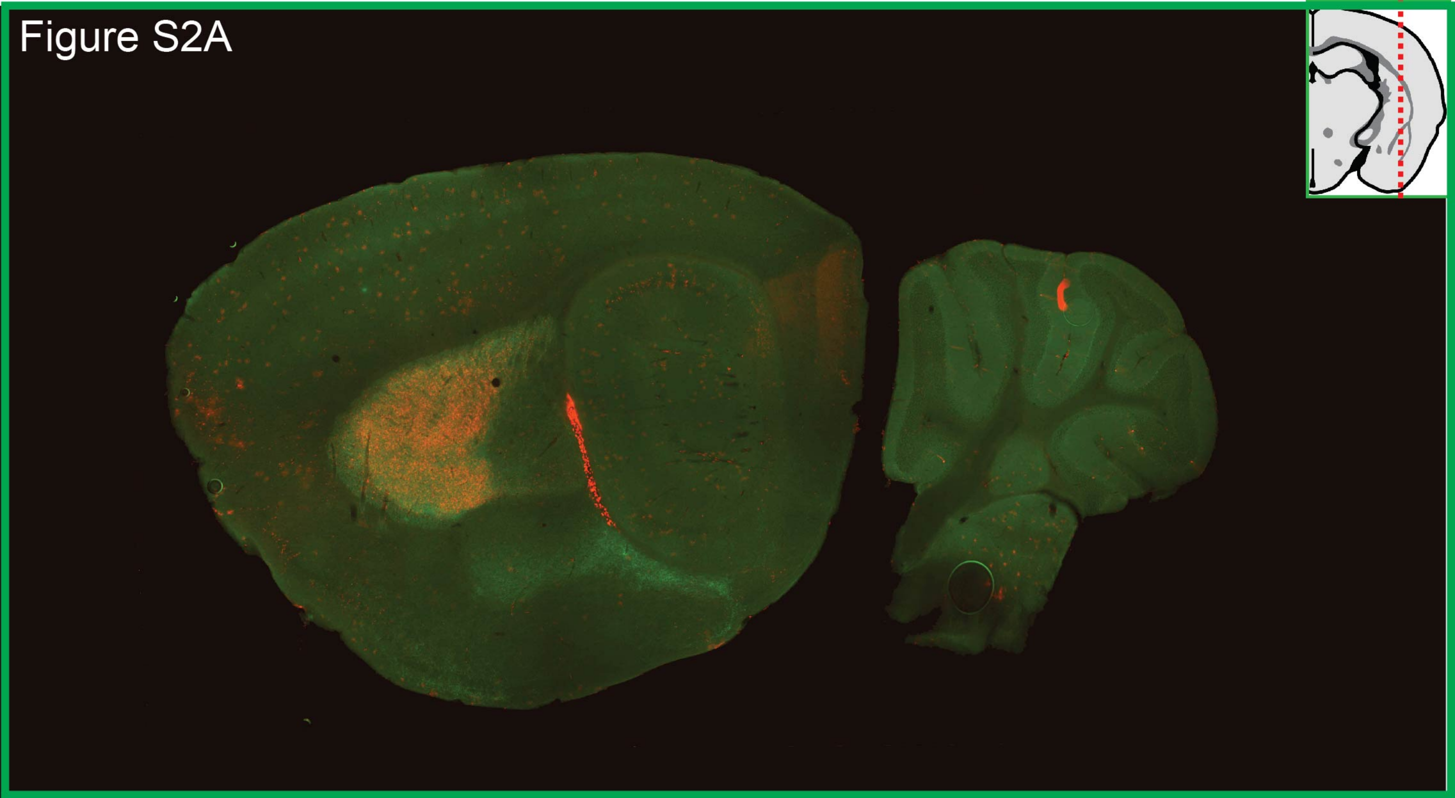

Figure S2B

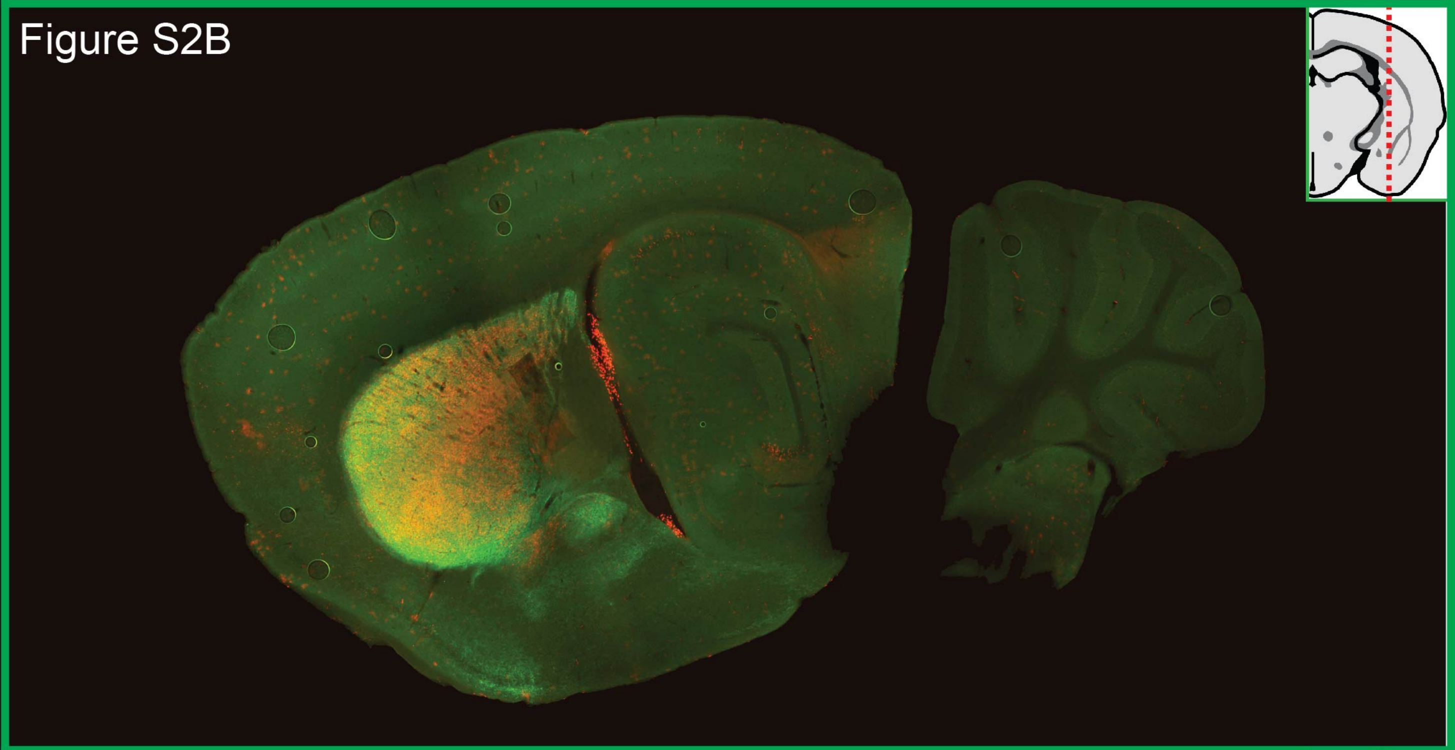

Figure S2C

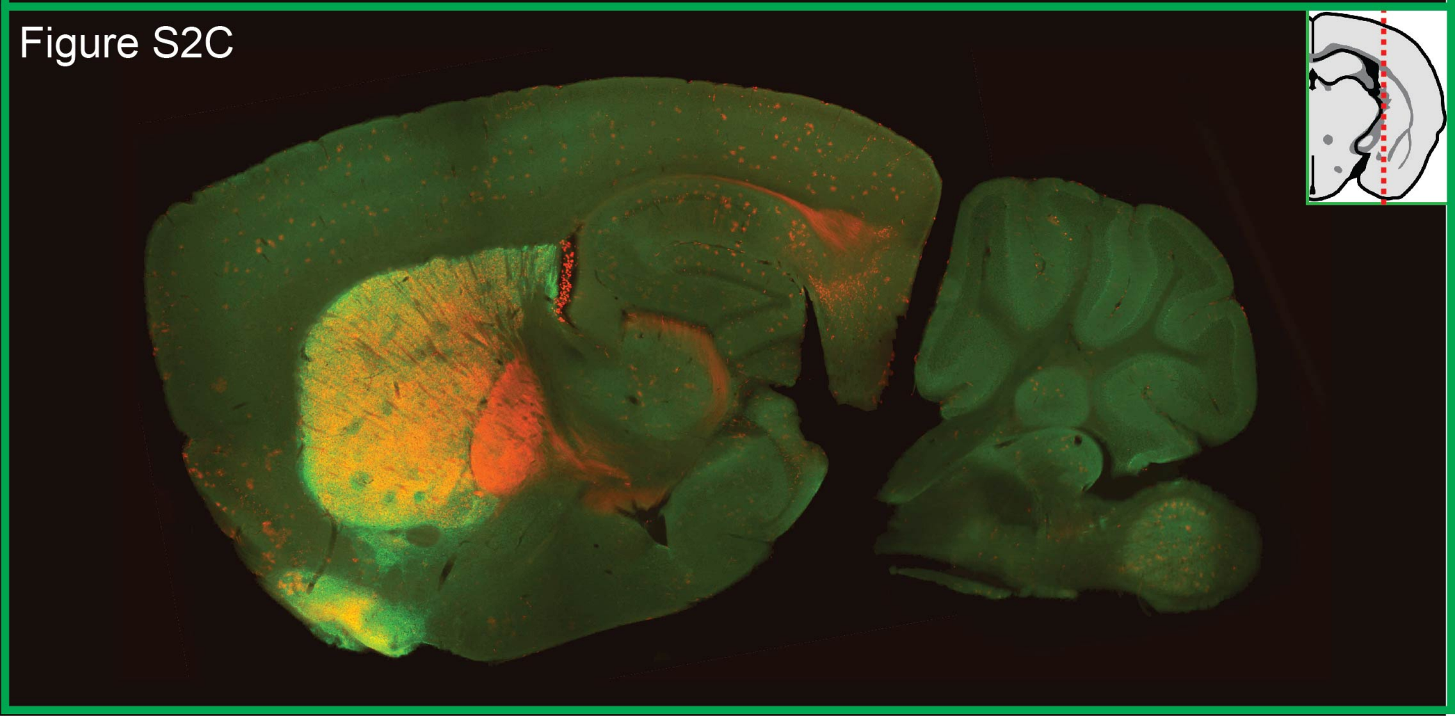

Figure S2D

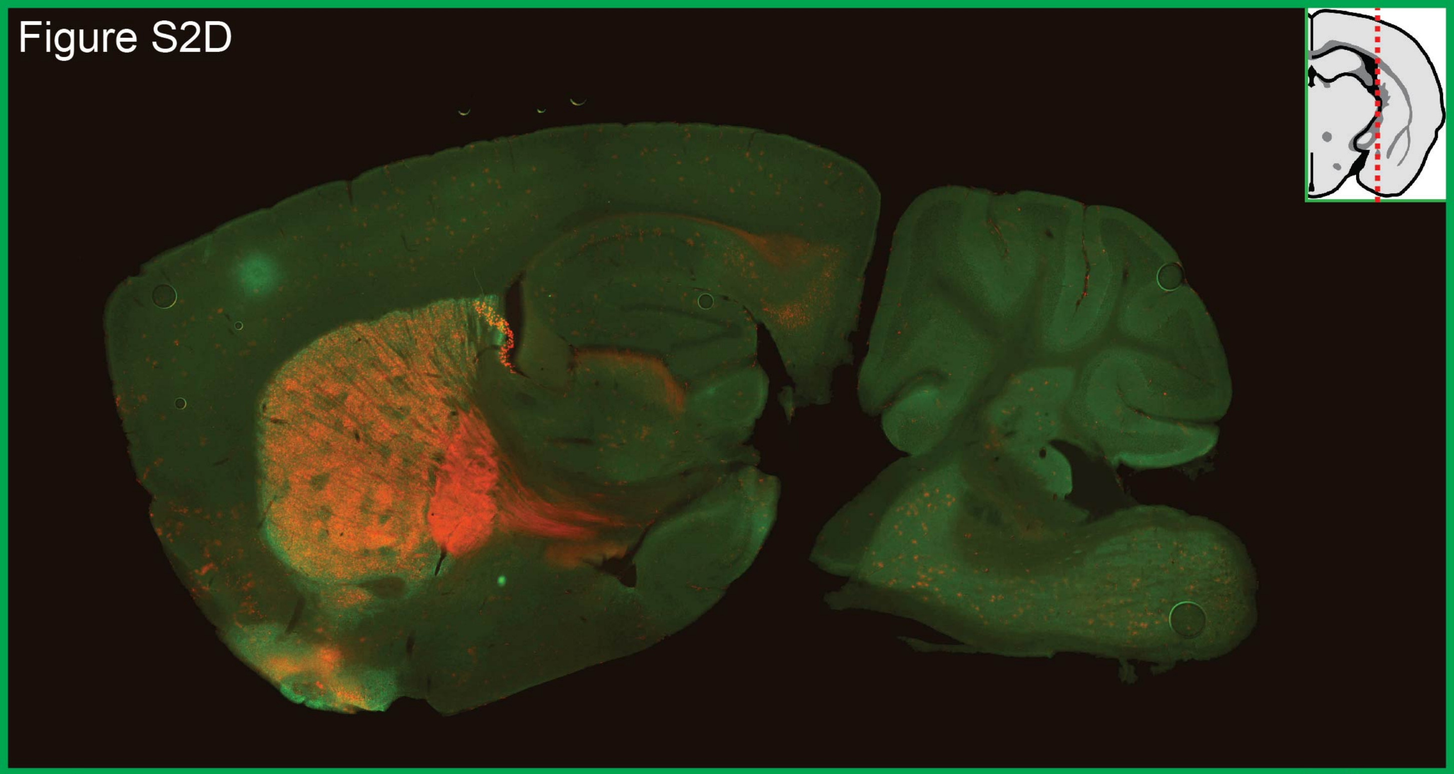

Figure S2E

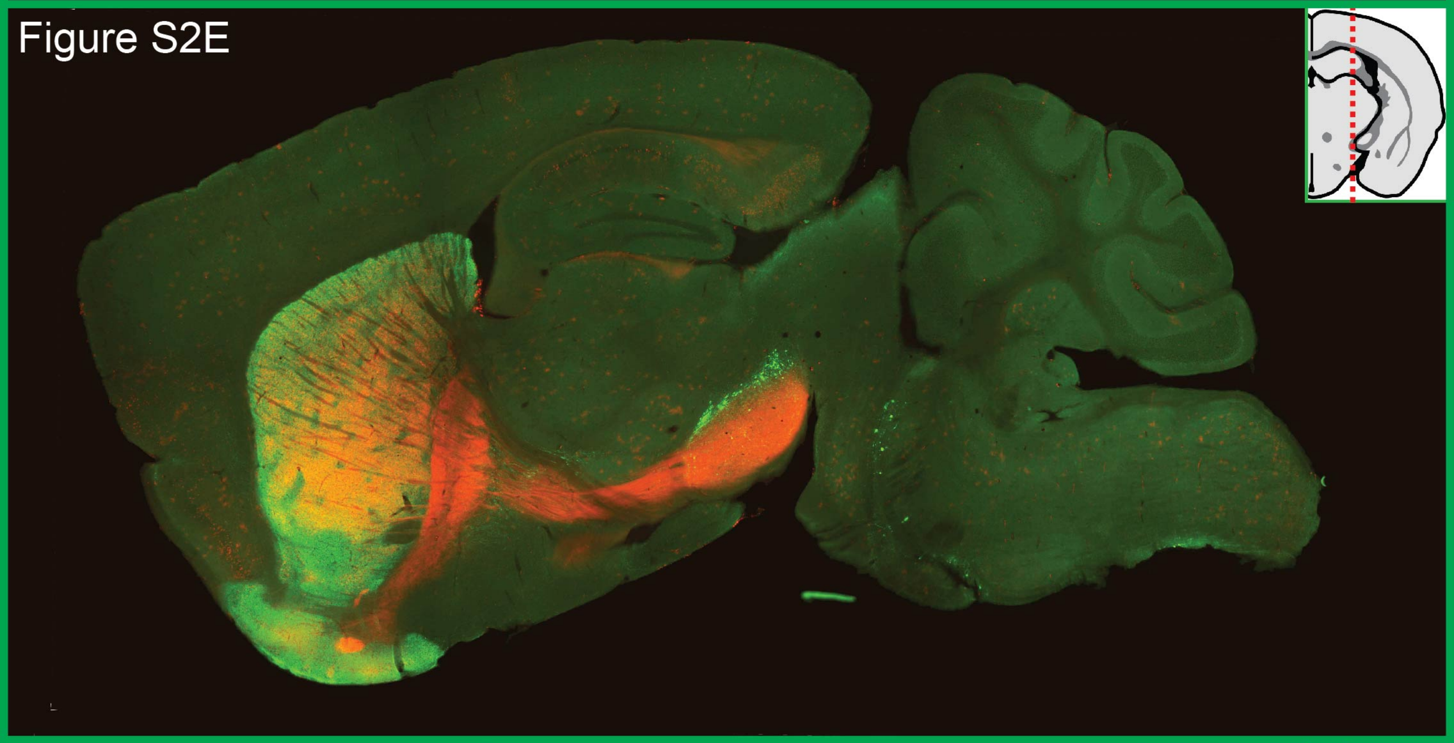

Figure S2F

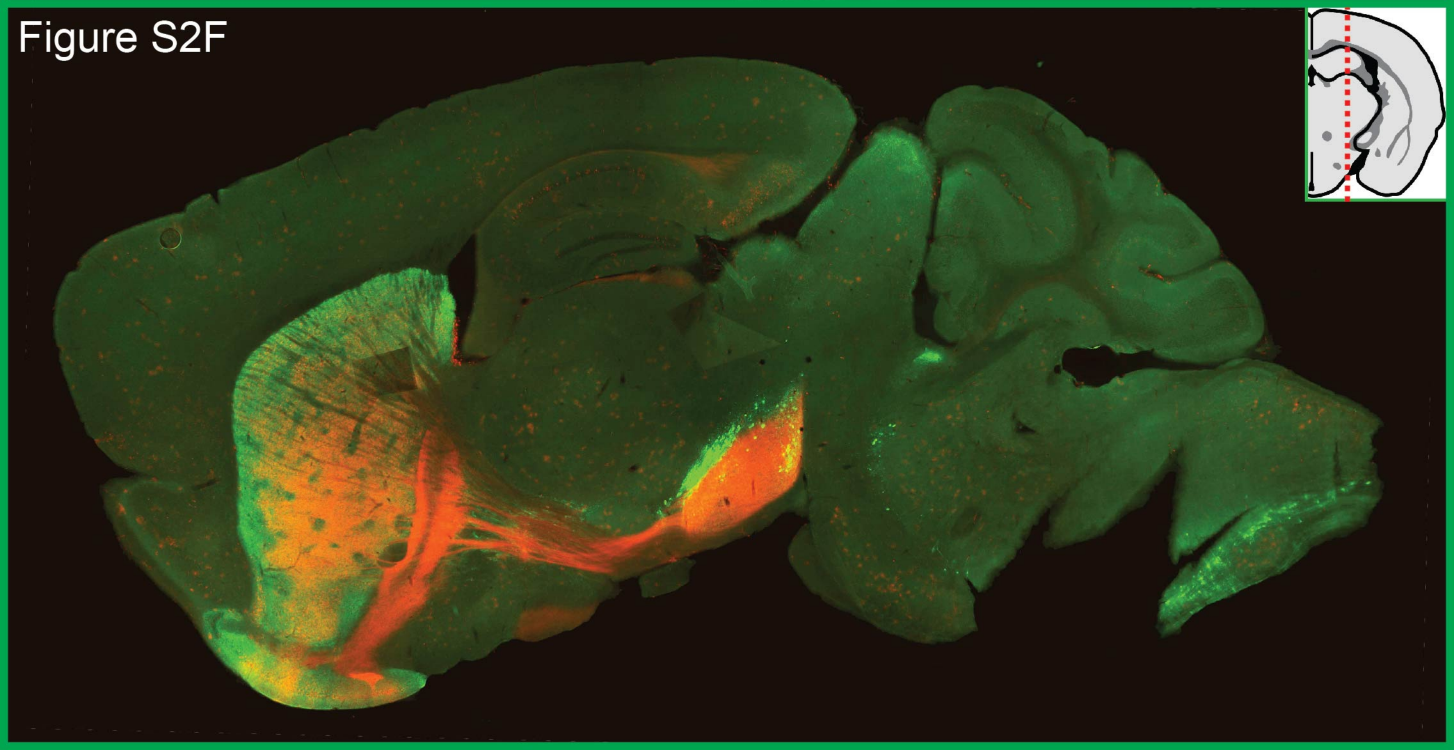

Figure S2G

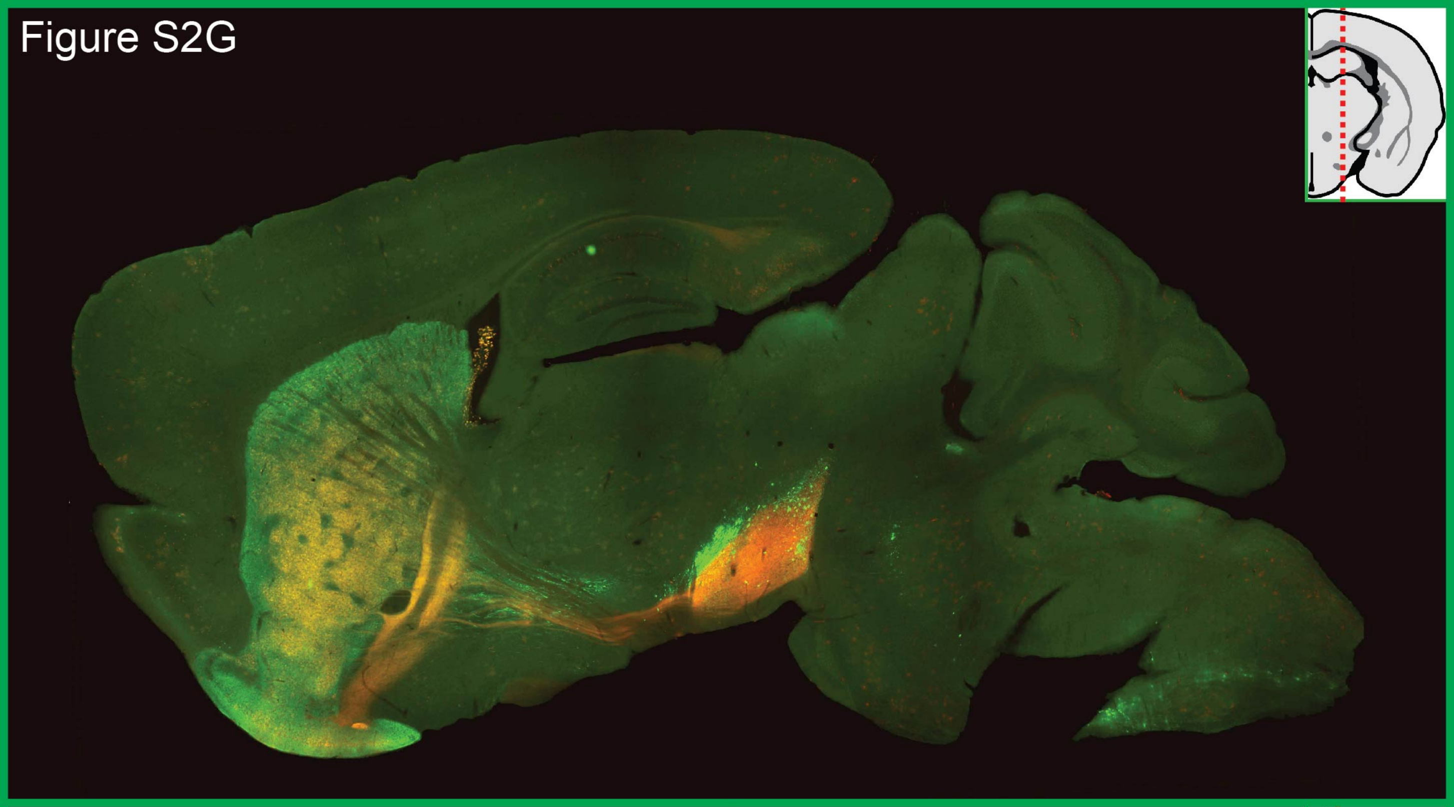

Figure S2H

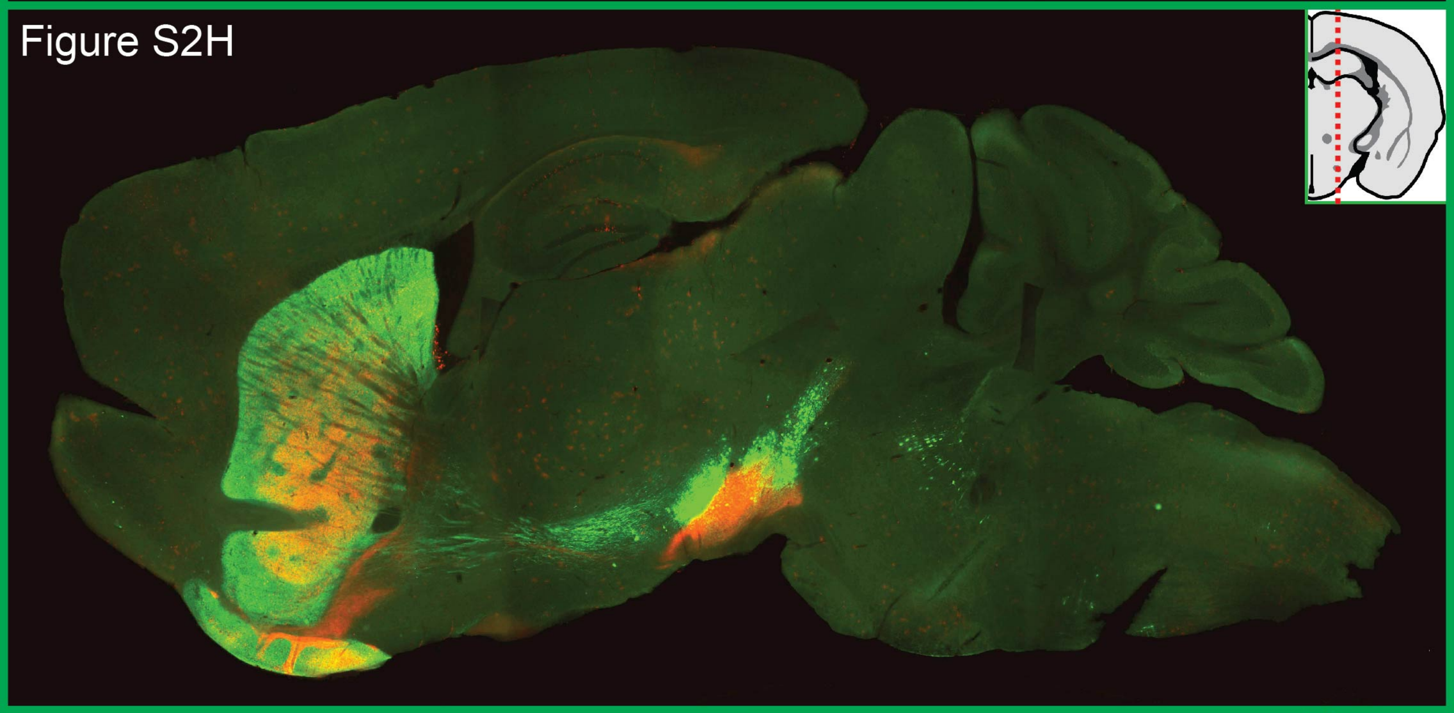

Figure S2I

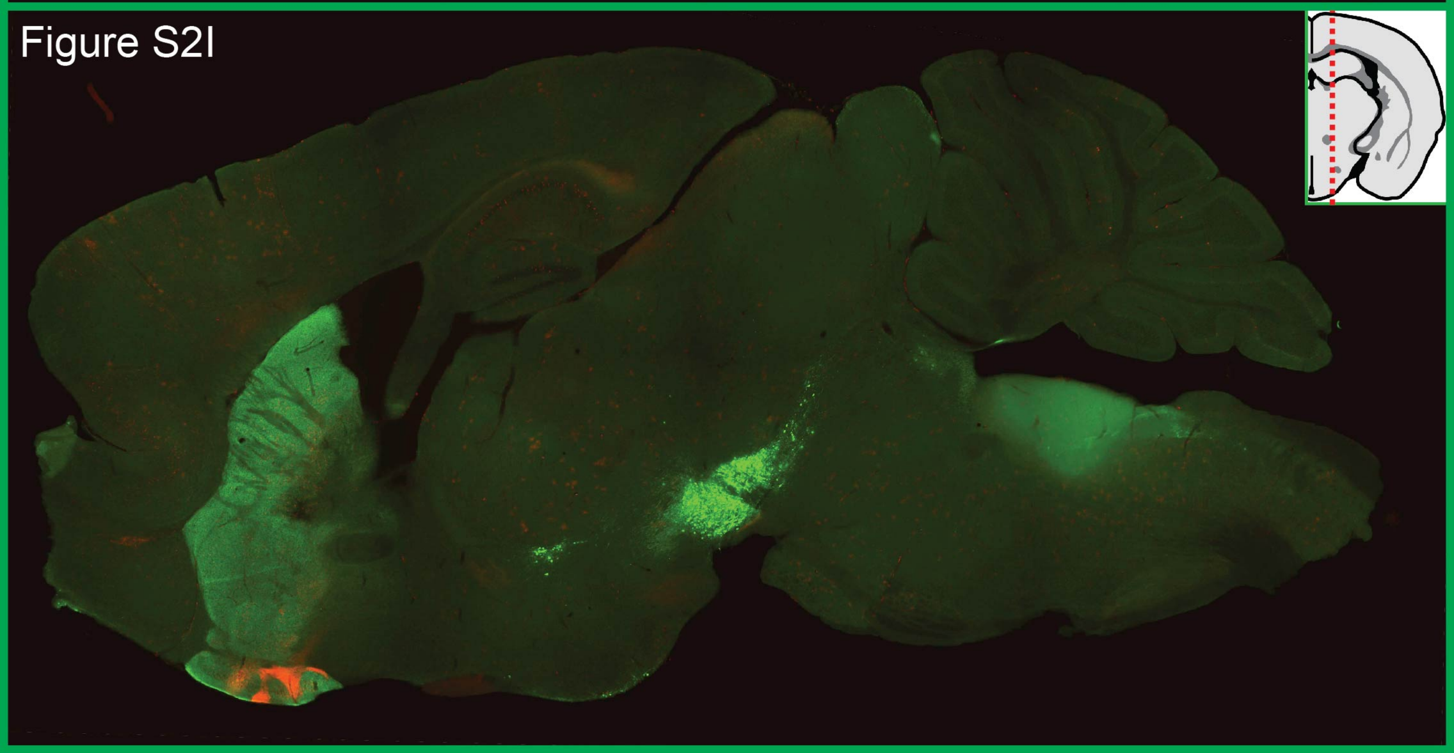

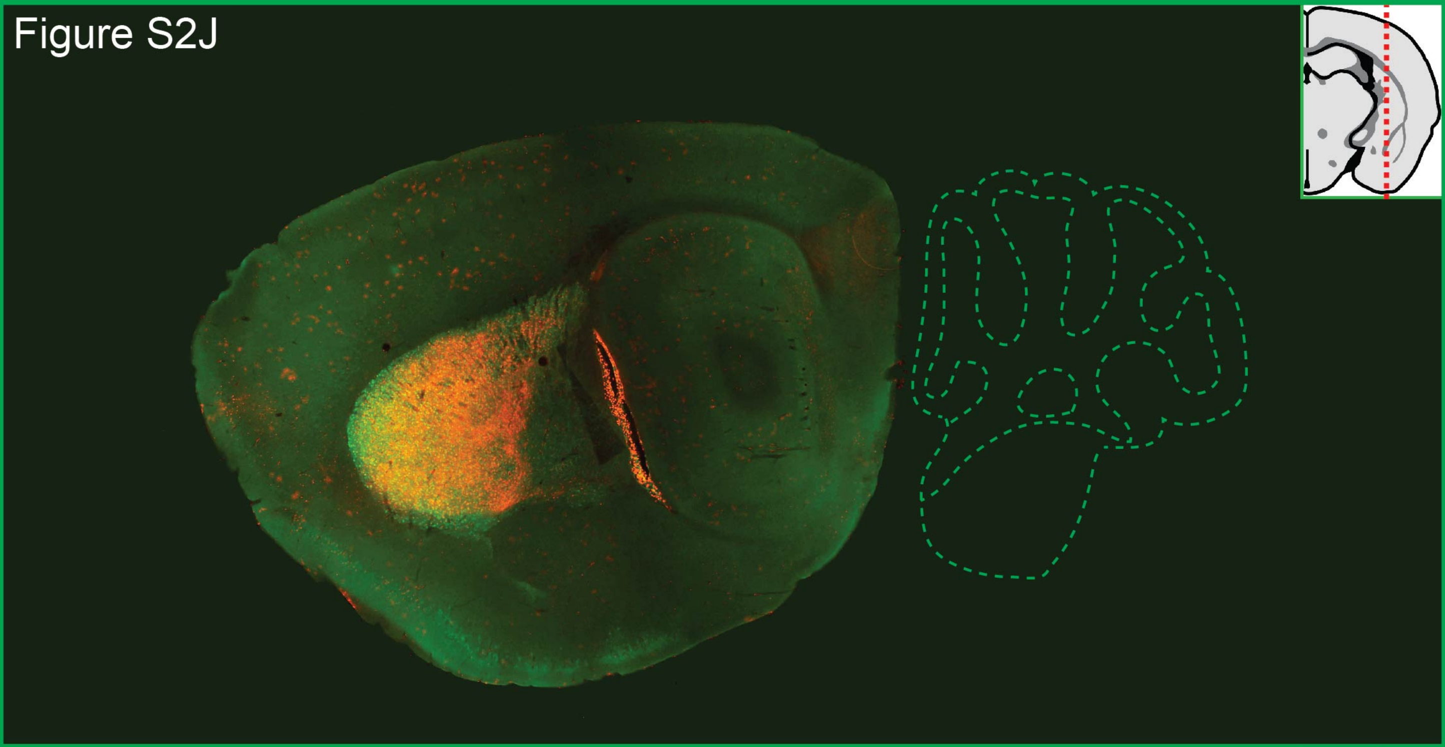
Figure S2J

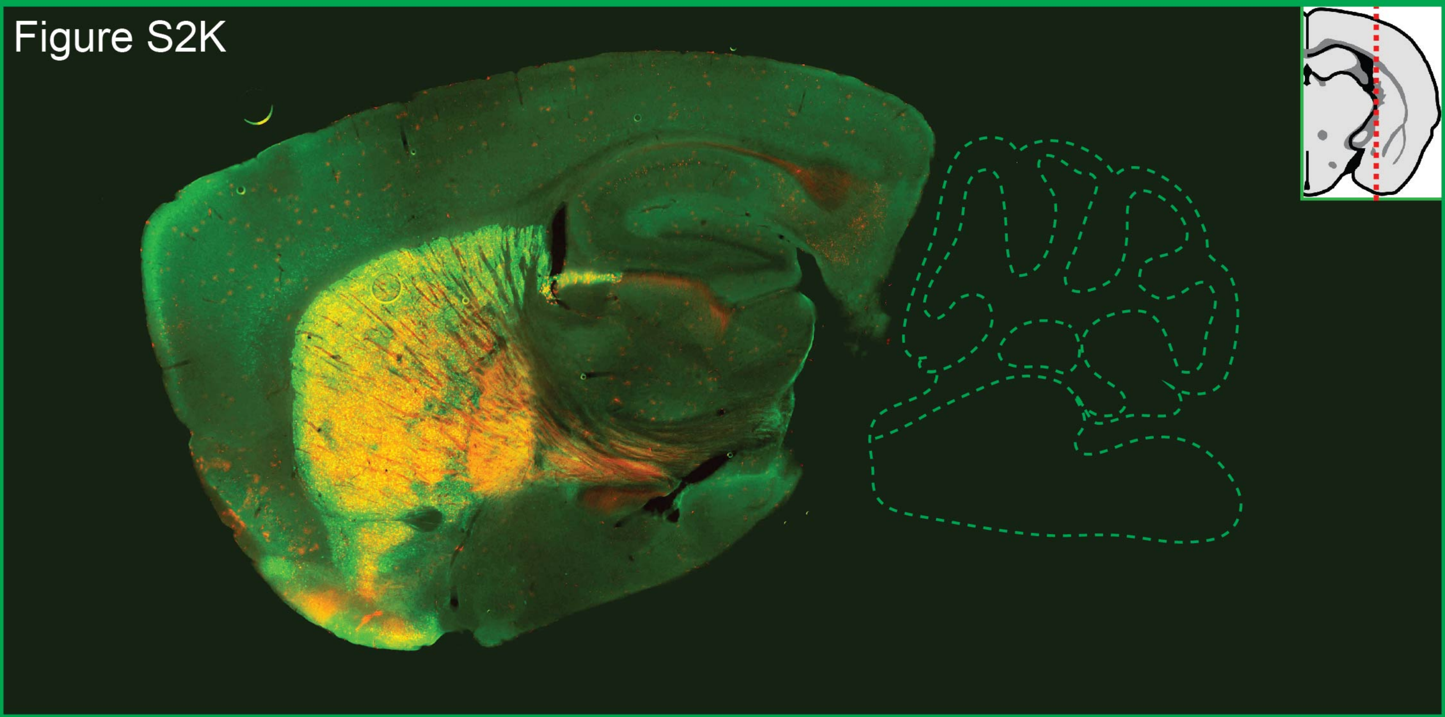
Figure S2K

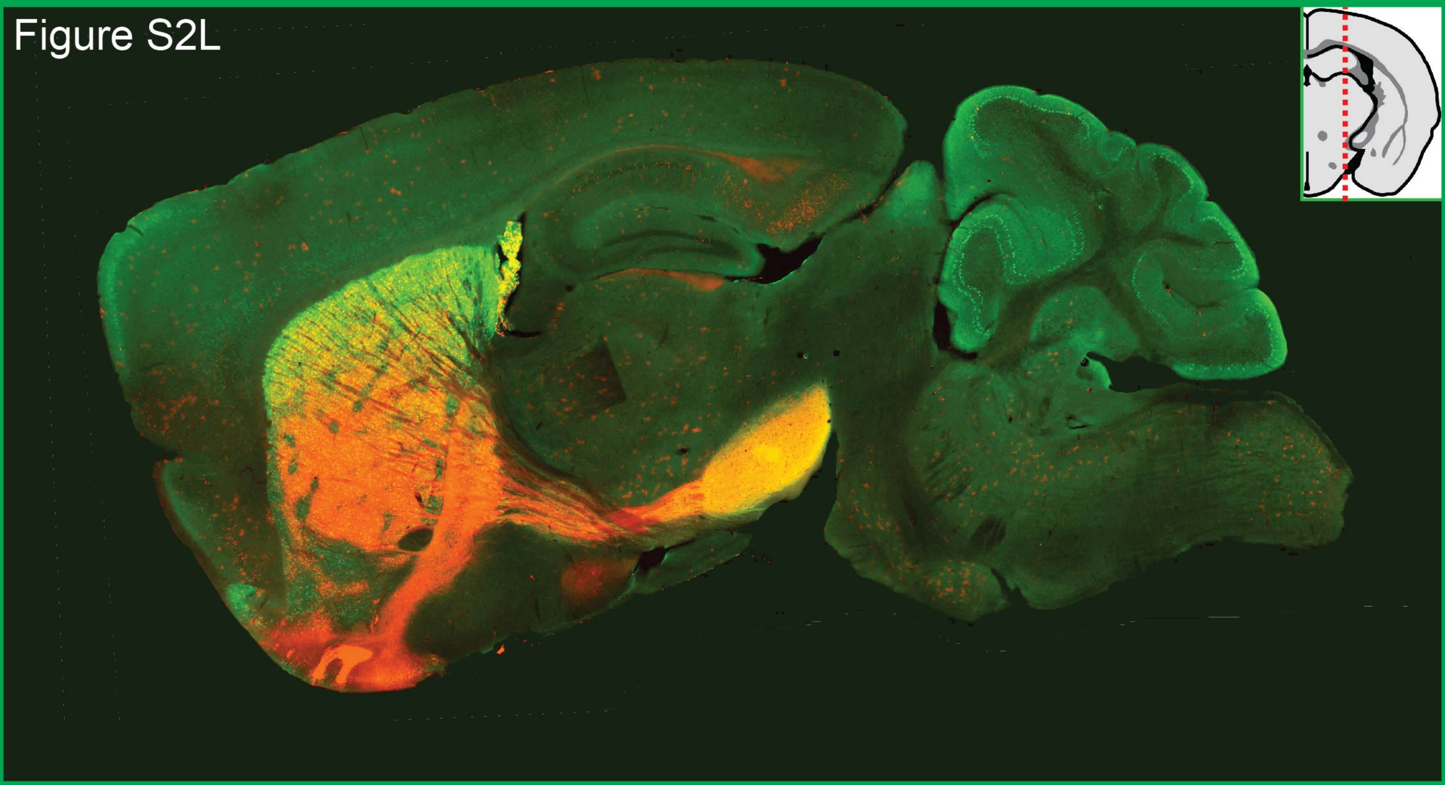
Figure S2L

Figure S2M

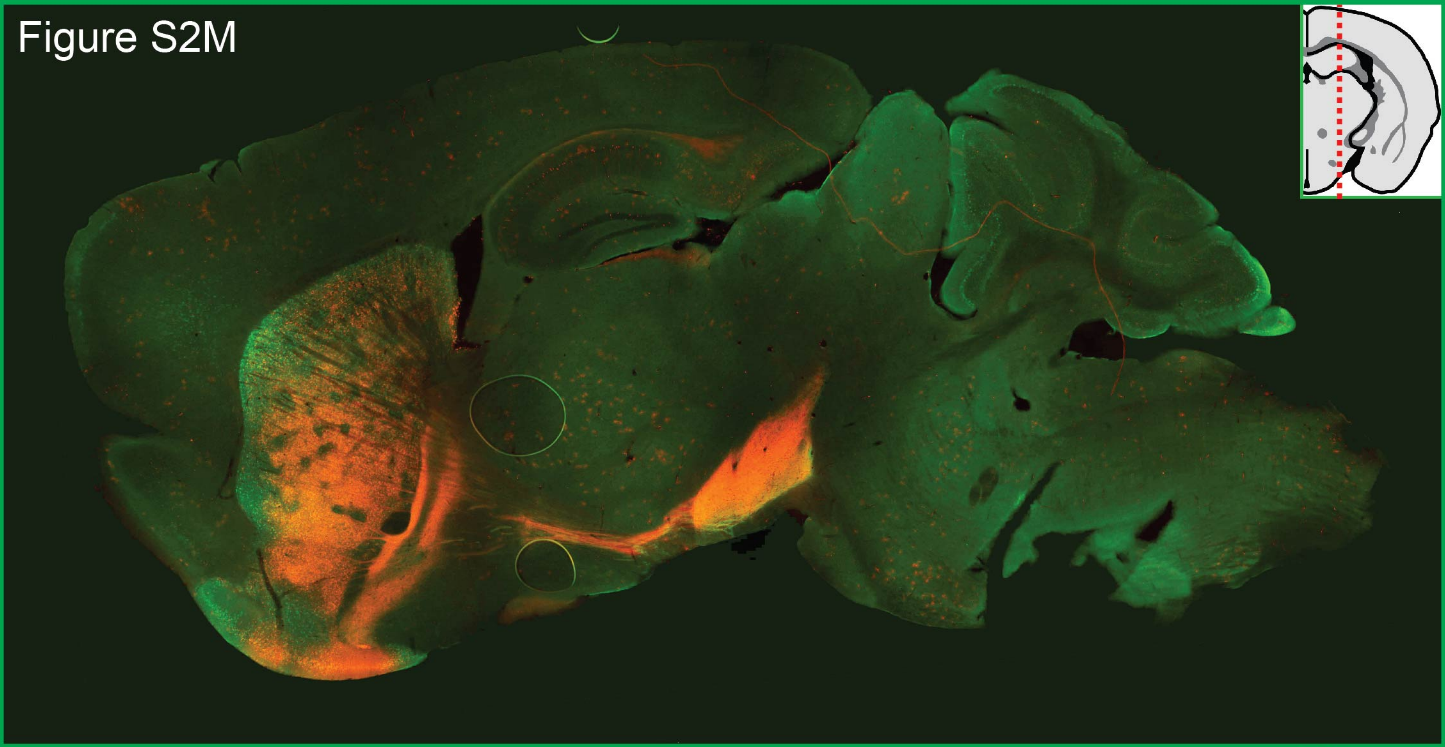

Figure S2N

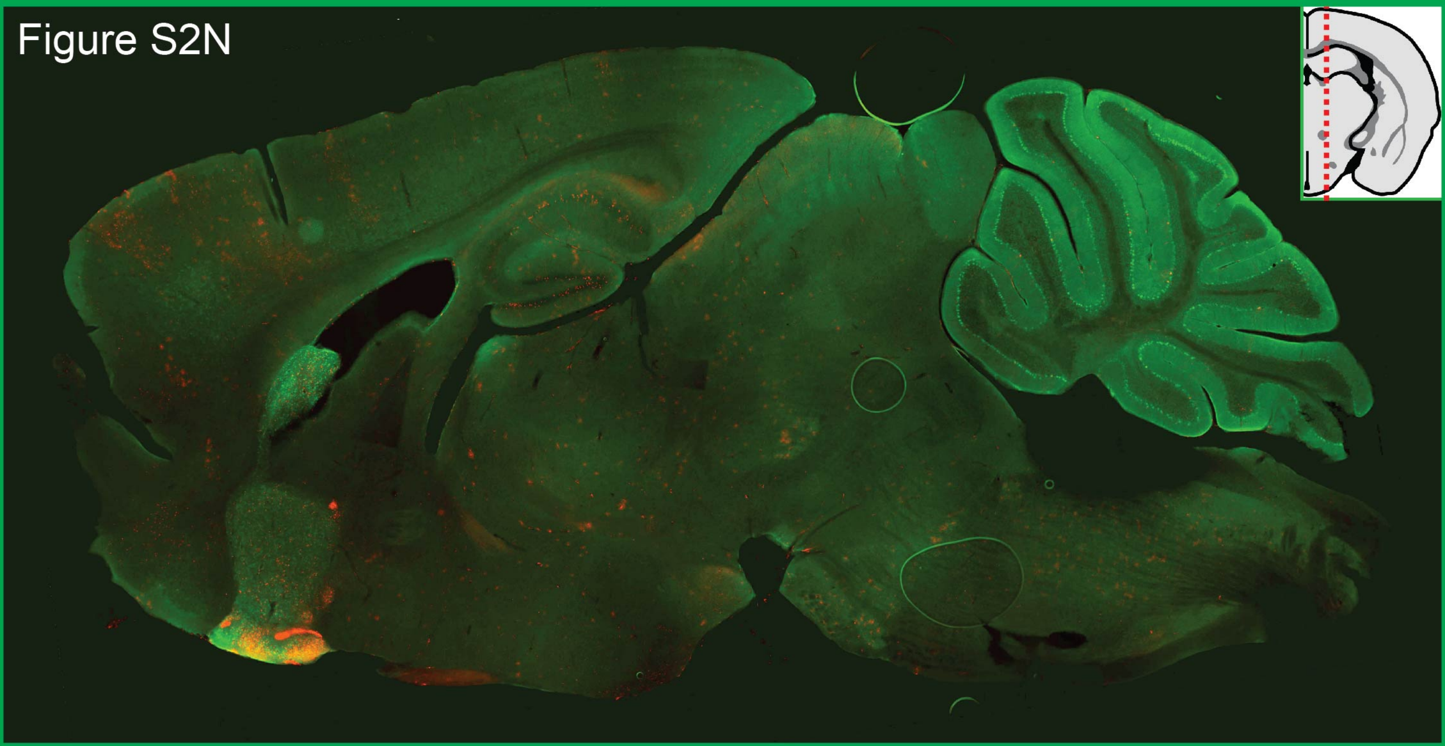

Figure S2O

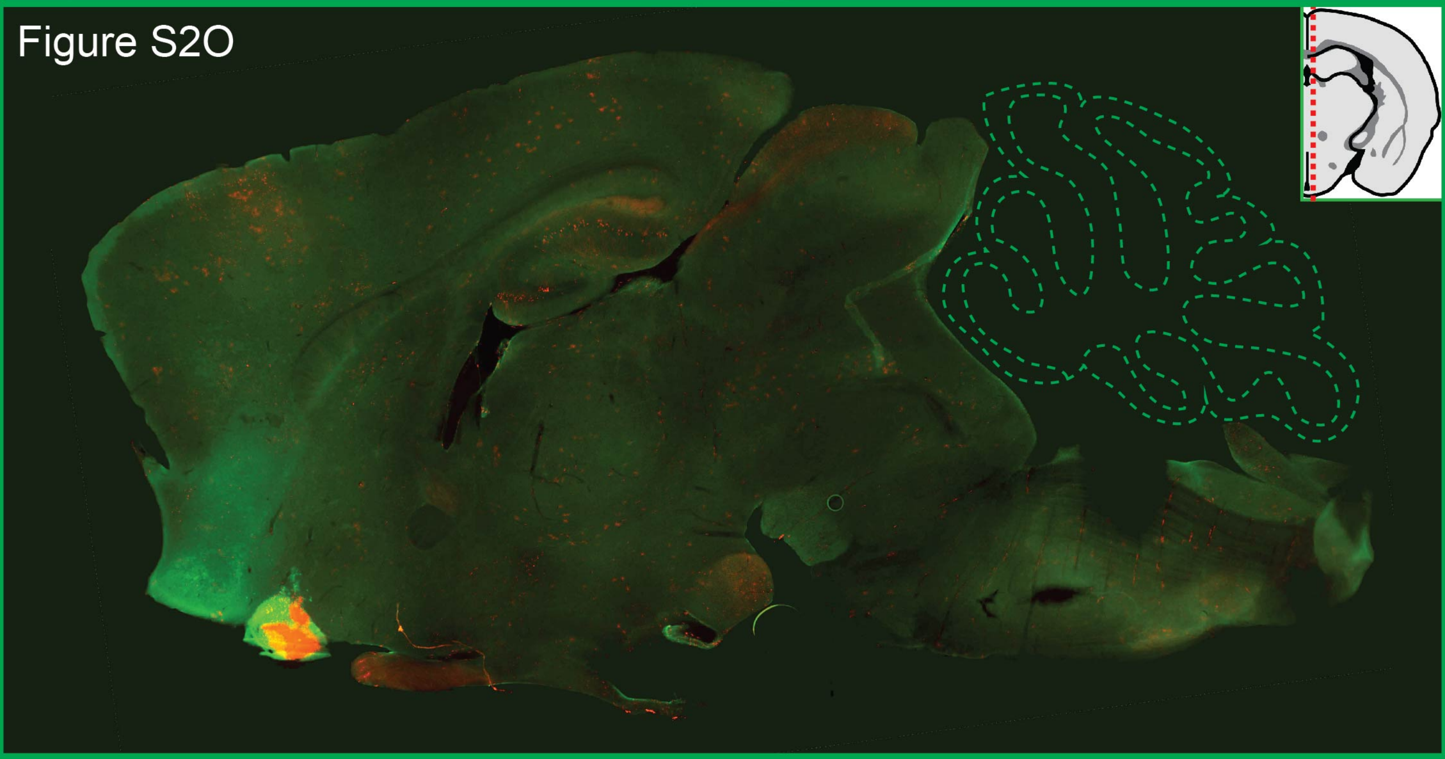

Figure S3

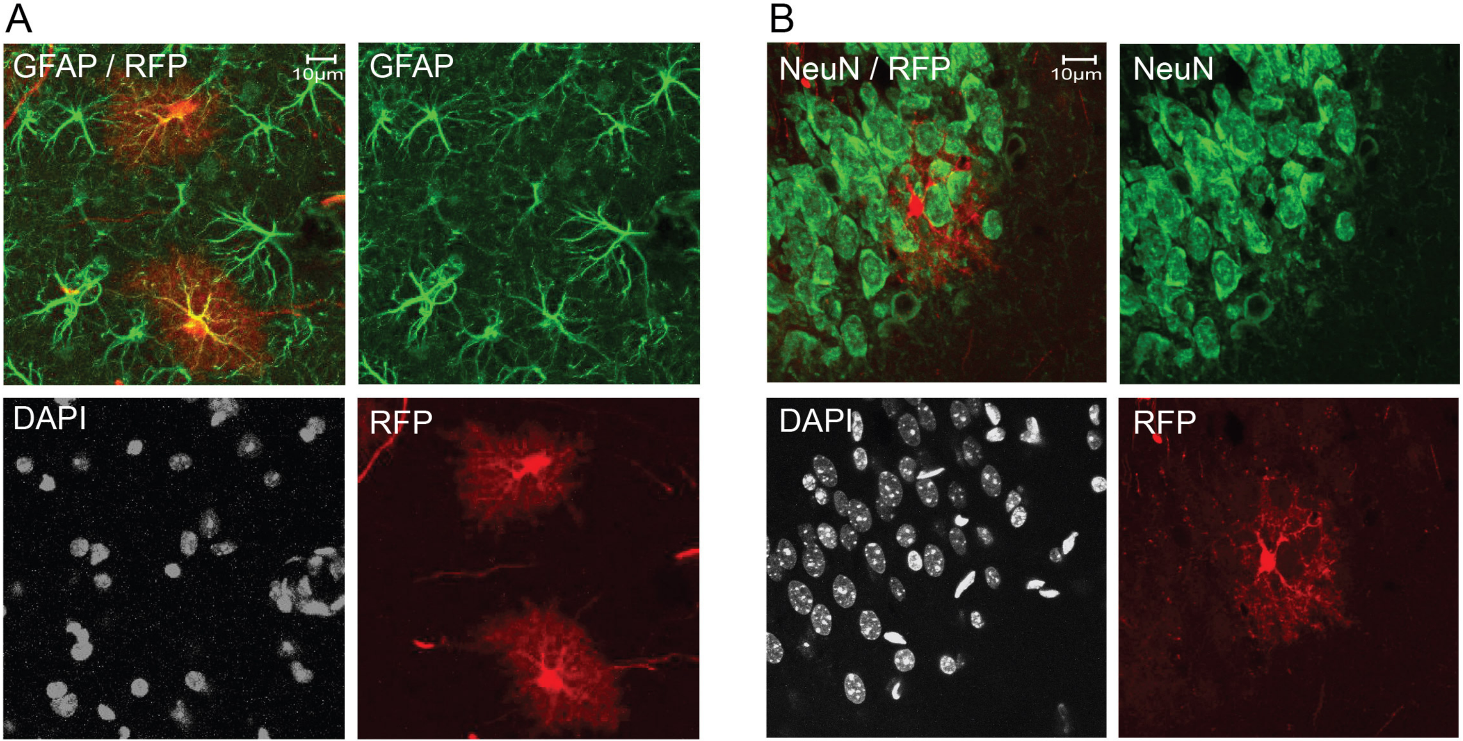

Figure S4

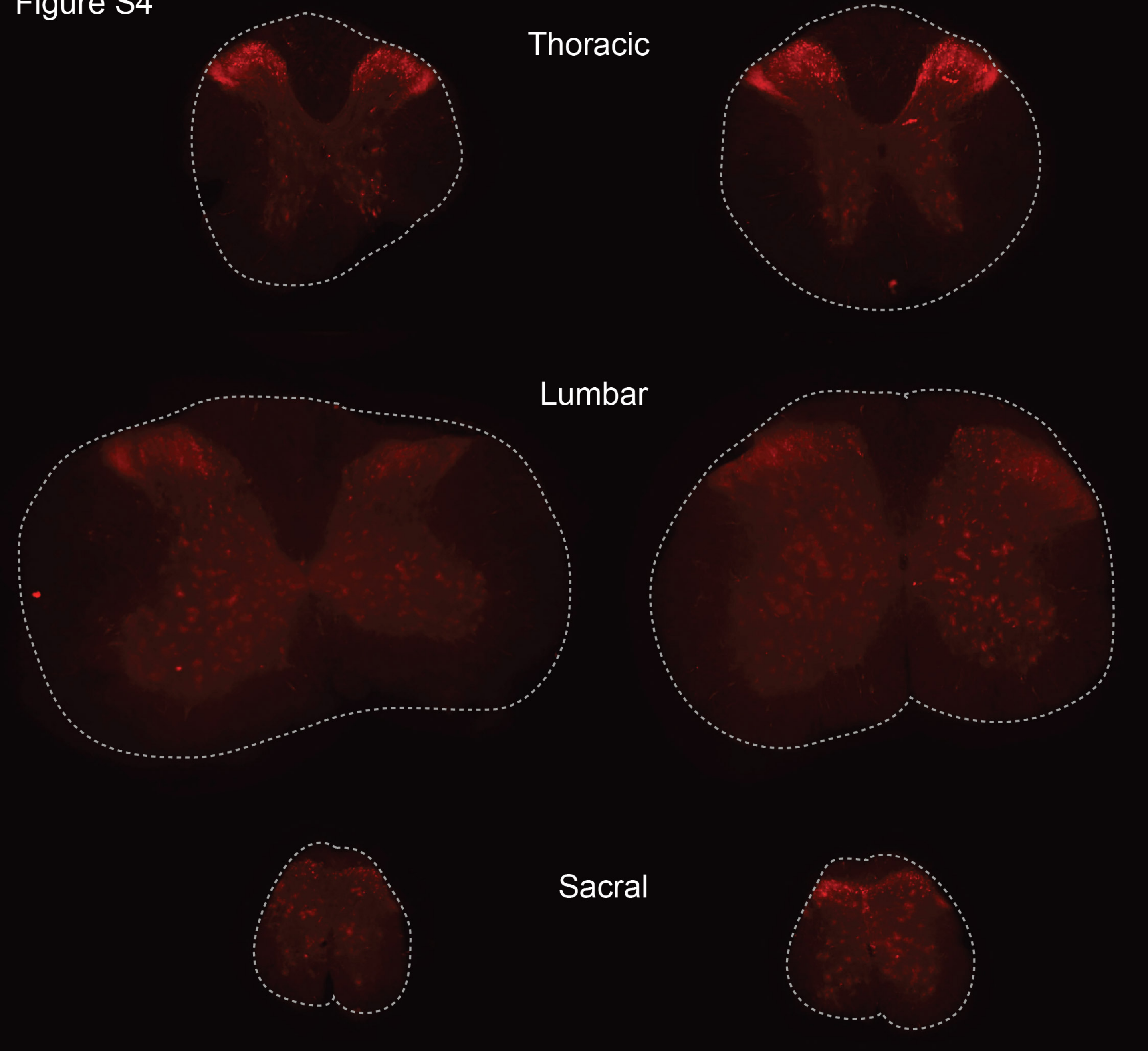

Figure S5

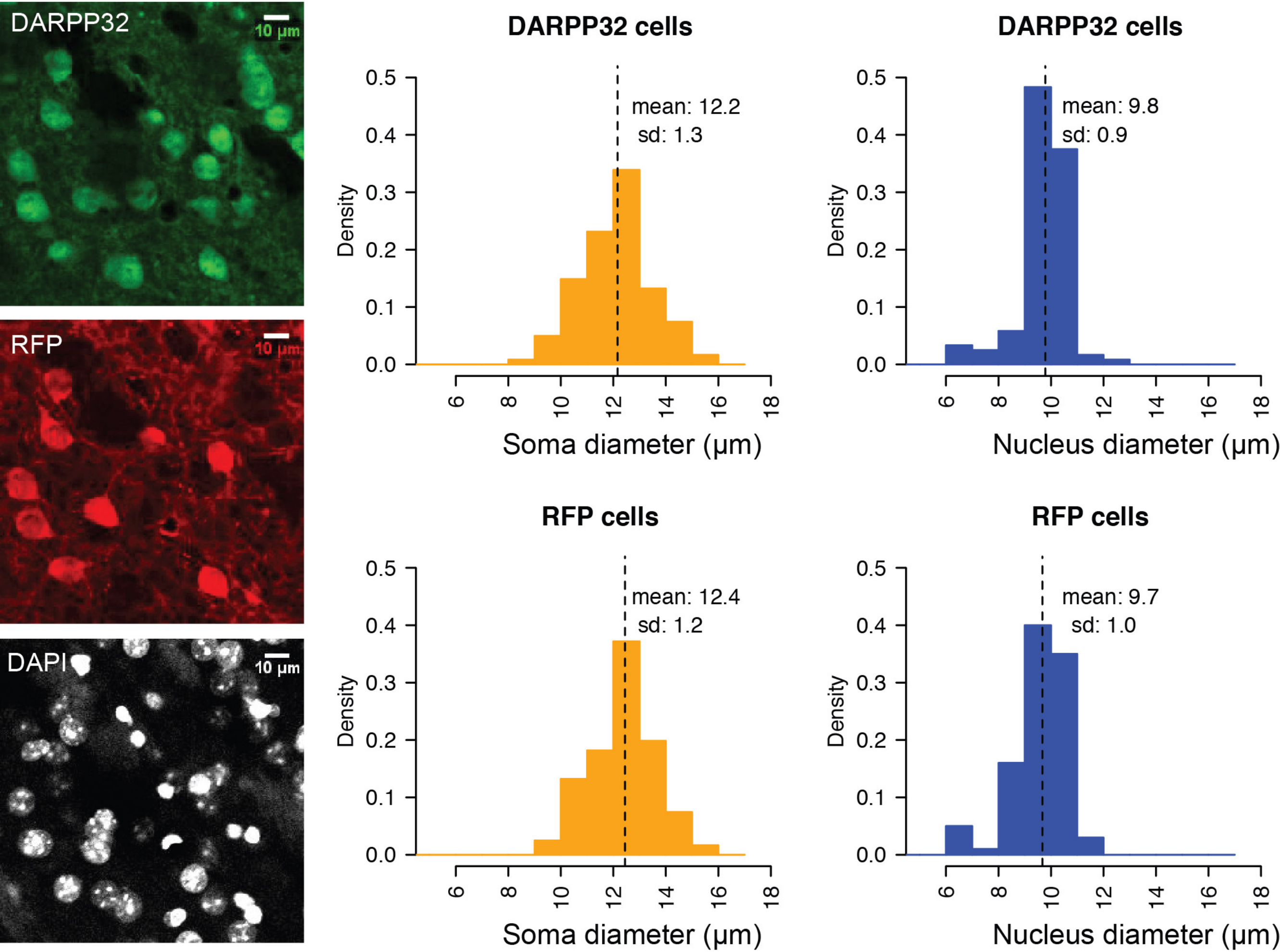
DARPP32
10 μm
RFP
10 μm
DAPI
10 μm
DARPP32 cells
mean: 12.2
sd: 1.3
Density
Soma diameter (μm)
DARPP32 cells
mean: 9.8
sd: 0.9
Density
Nucleus diameter (μm)
RFP cells
mean: 12.4
sd: 1.2
Density
Soma diameter (μm)
RFP cells
mean: 9.7
sd: 1.0
Density
Nucleus diameter (μm)

Figure S6

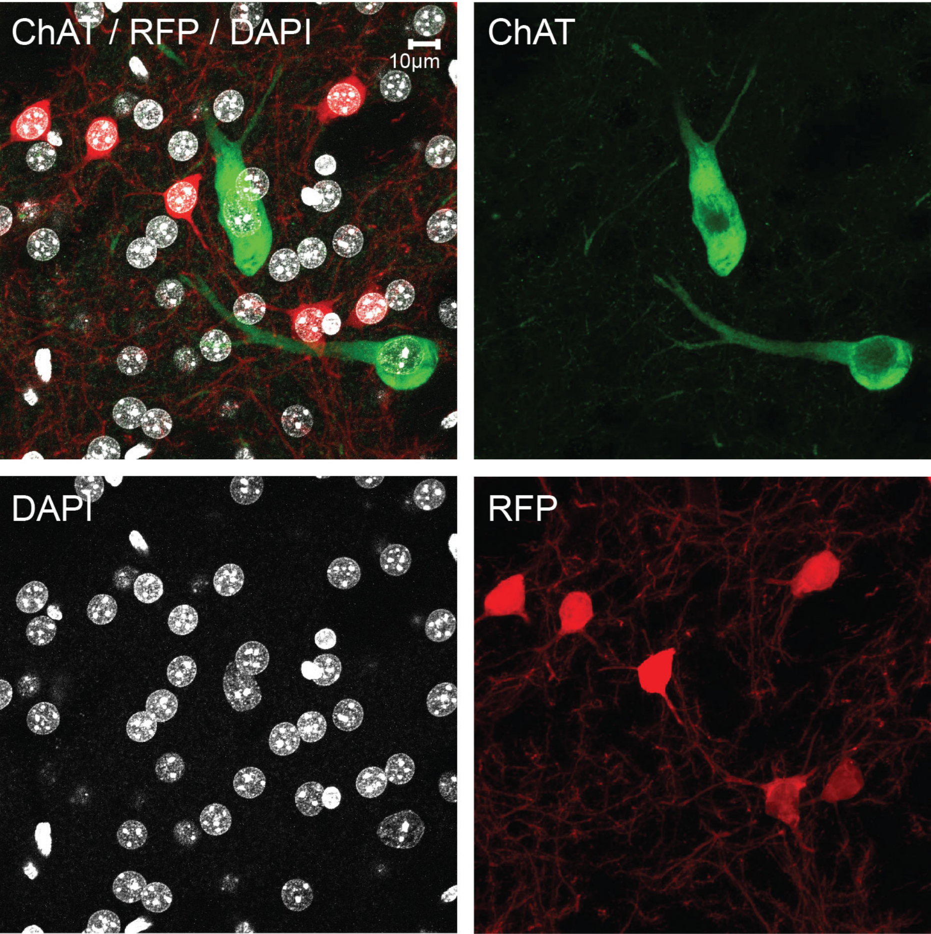

Figure S7

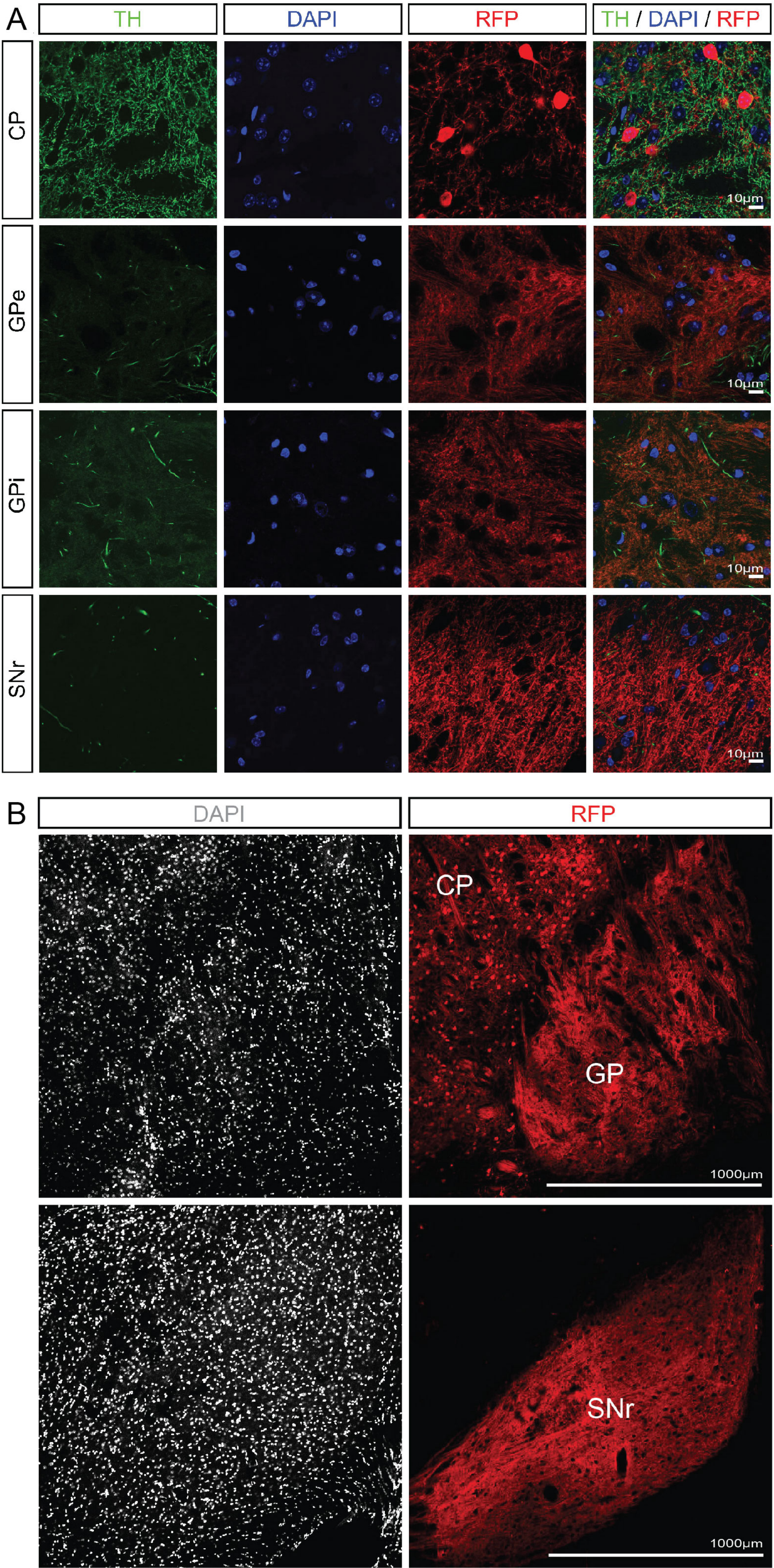
A
TH
DAPI
RFP
TH / DAPI / RFP
CP
GPe
GPi
SNr
10μm
10μm
10μm
10μm
B
DAPI
RFP
CP
GP
1000μm
SNr
1000μm

Figure S8

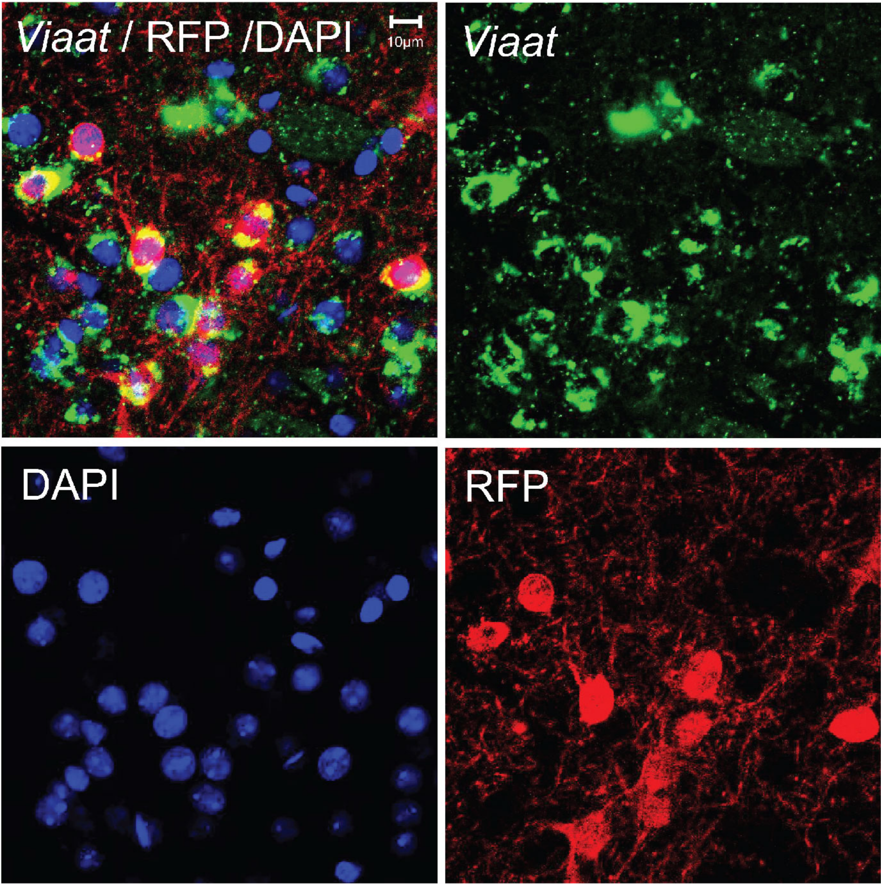

Figure S9

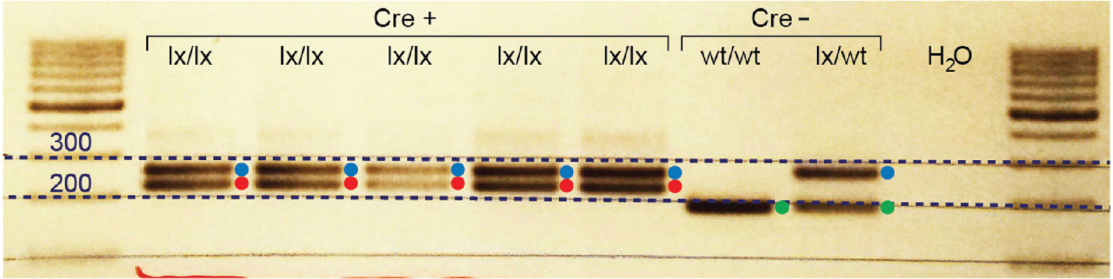

Supplement: Supplementary file 1 [file SupplementaryFigures.PDF]
